# Brown and white adipocyte like cells derived exosomes differentially regulate stemness, hormone secretion, apoptosis, and lipid metabolism in model cells

**DOI:** 10.1371/journal.pone.0355955

**Published:** 2026-09-01

**Authors:** Zeinab Ghesmati, Mohsen Rashid, Shabnam Fayezi, Effat Alizadeh, Masoud Darabi

**Affiliations:** 1 Department of Medical Biotechnology, Faculty of Advanced Medical Sciences, Tabriz University of Medical Sciences, Tabriz, Iran; 2 Department of Molecular Medicine, Faculty of Advanced Medical Sciences, Tabriz University of Medical Sciences, Tabriz, Iran; 3 University of Luebeck, Department of Gynecology and Obstetrics, University Fertility Center, Campus Luebeck, Luebeck, Germany; 4 University of Luebeck | Department of Hematology and Oncology, University Cancer Center Schleswig-Holstein (UCCSH) and University Hospital Schleswig-Holstein (UKSH), Campus Luebeck, Luebeck, Germany; Tarbiat Modares University, IRAN, ISLAMIC REPUBLIC OF

## Abstract

White and brown adipocytes show therapeutic potential, yet their exosomes mediated cell-specific effects remain poorly understood. This study aimed to assess the impact of conditioned media (CM) or exosomes derived from brown and white adipocyte-like cells (BALCs and WALCs, respectively) on four human cell types, including ovarian granulosa cells (OGCs), MCF-7 breast cancer cells, human adipose-derived stem cells (hADSCs), and human umbilical cord mesenchymal stem cells (hUCMSCs). Isolated hADSCs were differentiated into BALCs and WALCs using specific induction media. Corresponding exosomes were isolated from BALCs or WALCs derived CM and characterized. Human cell types—representing stem, somatic, and cancer cells—were treated with CM or exosomes. Cellular responses and lipidomic profiling were evaluated using molecular biology techniques and mass spectrometry, respectively. BALCs-derived exosomes significantly increased estradiol secretion from ovarian granulosa cells (p < 0.05). In MCF-7 cells, CM had a stronger pro-apoptotic effect than exosomes (>2-fold). In hADSCs, both CM and exosomes promoted osteogenic differentiation (2.3-fold) and reduced stemness markers proteins Oct4 and Sox2 (p < 0.05). When treated with BALCs-derived exosomes, hADSCs displayed the highest number of differentially expressed lipids, primarily upregulated hits. Pathway analysis indicated that BALCs-derived exosomes promoted lipid storage and membrane remodeling in hADSCs, enhanced energy metabolism in granulosa cells, reduced fatty acid oxidation in MCF-7 cells, and decreased lipid storage in hUCMSCs. Our results highlight a potential modulatory role of adipocyte-derived exosomes in intercellular communication. The findings of this study may be applicable in regenerative medicine, metabolic regulation and cancer therapeutic approaches.

## Introduction

Adipose tissue is a large and complex group of active endocrine organs in the body [1]. It is also recognized as a unique tissue in regenerative medicine [2]. Through its various adipocytes, adipose tissue produces and secretes hormones, adipokines, as well as other small molecules that play crucial roles in numerous physiological processes [3]. Broadly, two types of adipose tissue—white adipose tissue (WAT) and brown adipose tissue (BAT)—differ morphologically and functionally. Shortly after birth, development of WAT starts [4], typically characterized by an ivory or yellowish color, contains unilocular (large single) lipid droplets (LDs) with flattened nuclei, limited mitochondria, and low or no expression of uncoupling protein 1 (UCP1) [1]. WAT accounts for >95% of the fat mass and captures metabolic substrates from blood plasma such as free fatty acids as well as glucose and converting them into triglycerides (TGs) through lipogenesis [5]. As ubiquitous organelles, LDs store and supply lipids for various cellular functions [6,7]. Perilipins (PLINs) refer to a family of LD cytoplasmic surface proteins [8]. In mammals, five PLIN proteins have been identified and numbered in order of their discovery (PLIN1–5) [7,8]. Compared with BAT, PLIN1 is found more abundant in WAT [7].

Before birth, BAT forms to protect the newborn from cold [1]. In humans, with age, BAT remains only around deeper organs representing 1–2% of total adipose tissue [1,9]. BAT appears macroscopically brownish compared to WAT due to its higher mitochondrial content [10,11]. Morphologically, BAT is composed of spherical brown adipocytes containing many multilocular (small) LDs with heterogeneous in size and high levels of UCP1 expression [1,12]. As a highly metabolic tissue, BAT can convert a variety of substrates, including glucose and fatty acids into heat through mitochondrial oxidative phosphorylation through UCP1-mediated uncoupled respiration to support non-shivering thermogenesis [9].

BAT and WAT are not merely considered heat producers and consumers. Both white and brown adipocytes secrete various molecules packaged in extracellular vesicles (EVs) [13]. Exosomes, a subclass of EVs ranging from 30–160 nm in diameter, carry diverse cargo, including cytosolic and cell-surface proteins, lipids, amino acids, metabolites, as well as other bioactive molecules [14]. These vesicles facilitate efficient intercellular communication both locally and systemically [15,16], with their biological functions determined by their cargo [16,17]. EVs are emerging as promising cell‑free therapeutic tools, thereby minimizing the linked to direct cell administration. While EVs derived from white and brown adipocytes exhibit therapeutic potential, their cell‑specific effects remain poorly characterized. Thus, a deeper comprehension of paracrine mechanisms is critical for developing new therapeutic strategies.

Obesity, diabetes, cardiovascular diseases, cancer and infertility are closely linked to adipose tissue dysfunction [1]. Thus, understanding the interactions between adipocytes and other tissues/organs is essential for uncovering the mechanisms underlying these disorders [18]. Based on our previous studies, we selected four human cell types as in vitro models to examine interorgan interactions: ovarian granulosa cells (OGCs), which are essential for oocyte maturation and fertility [19,20]; MCF-7 cells, a widely utilized breast cancer model with unclear pathology [21]; and human adipose-derived stem cells (hADSCs) and human umbilical cord mesenchymal stem cells (hUCMSCs), which are valued in regenerative medicine for their high abundance as well as plasticity [22,23]. We assessed how paracrine derivatives from mesenchymal stem cell (MSC)–differentiated brown adipocyte-like cells (BALCs) and white adipocyte-like cells (WALCs) influence cell phenotypes and lipid profiles.

## Materials and methods

**Fig 1:**
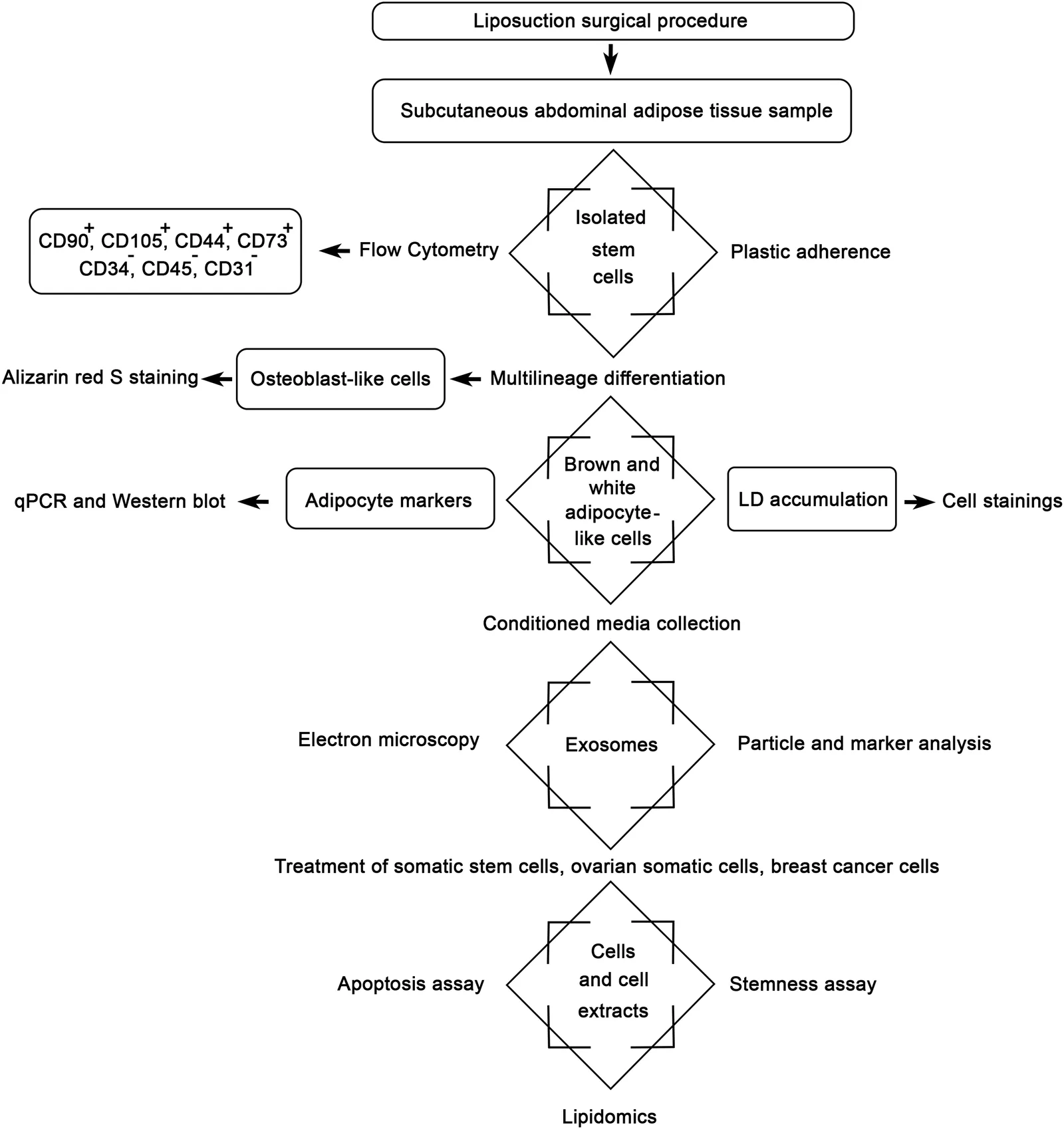
A flowchart of the experimental workflow. Human adipose-derived stem cells were differentiated into brown and white adipocyte-like cells. Conditioned media (CM) were applied for the isolation of exosomes. The isolated exosomes, along with crude CM, were used as treatment agents for adipose-derived stem cells, umbilical cord mesenchymal stem cells, somatic ovarian granulosa cells, and MCF-7 breast cancer cells. LD: Lipid droplets.

A flowchart outlining the research process is presented in Fig 1.

### Ethics statement

This study was undertaken in accordance with the Declaration of Helsinki and was approved by the Research Ethics Committees of the Vice-Chancellor in Research Affairs, Tabriz University of Medical Sciences, under approval number IR.TBZMED.VCR.REC.1400.135 on June 28, 2021. Written informed consent was obtained from all participants. We collected subcutaneous abdominal adipose tissue from five normal female (age < 30) along cesarean section, from Woman ‘s Alzahra Hospital (Tabriz, Iran). The participants were informed that their participation was voluntary and that they could withdraw from the study at any time without any adverse consequences. In this study, there was no minor, and as such parental or guardian consent was not required. No waiver of informed consent was requested or granted by the ethics committee. The recruitment of participants and the collection of human samples for this study were conducted from June 28, 2021, to July 3, 2023.

### Isolation and culture of hADSCs

Human adipose tissue obtained from the abdominoplasty of a donor was minced, rinsed with phosphate-buffered saline (PBS), and digested with 0.1% collagenase type I at 37°C for 45–60 min. The digestion was terminated with low-glucose DMEM (Gibco, MD) + supplemented with 10% FBS, and the suspension was centrifuged to isolate the stromal vascular fraction. The cells were cultured in medium supplemented with 20 ng/mL bFGF and EGF. Non-adherent cells were removed after 24 h. The adherent hADSCs were expanded and passaged at 80–90% confluency using trypsin-EDTA. Cells from passages 3–4, which demonstrated high proliferation and colony-forming ability, were employed for experiments.

### Characterization of isolated hADSCs

#### Flow cytometry analysis.

hADSCs (80–90% confluency) were characterized via flow cytometry. Briefly, trypsinized cells (2 × 10^5^) were washed and incubated for 30 min at 4°C with APC-, PE-, FITC-, or PerCP-conjugated antibodies against CD90, CD105, CD44, CD73, CD34, CD45, and CD31. Following incubation, the cells were washed and analyzed using a BD FACSCalibur flow cytometer coupled with CellQuest Pro software. Isotype-matched IgG controls were included for each antibody.

#### Multilineage differentiation assay of hADSCs.

The ability to undergo multipotent differentiation, a hallmark of MSC identity, was assessed in the isolated hADSCs. hADSCs were induced to differentiate into osteogenic and adipogenic lineages using the respective induction media.

#### Osteogenic differentiation.

To induce osteogenic differentiation, hADSCs were cultured in osteogenic medium for 3 weeks, containing 0.1 μM dexamethasone, 50 μM L‑ascorbic acid 2‑phosphate, and 10 mM β‑glycerophosphate (Sigma‐Aldrich, MO). The medium was refreshed twice per week. After 21 days, the differentiated cells were fixed in 10% formalin at 4°C for 1 h and stained with 2% Alizarin Red S (pH 4.2) to detect calcium deposits in mineralized nodules.

#### Adipogenic differentiation: brown and white adipocyte-like cells.

MSCs can differentiate into BALCs and WALCs through adipogenesis [1]. In order to induce BALC differentiation, hADSCs were cultured for 3 weeks in medium containing 0.5 mM 3-isobutyl-1-methylxanthine (IBMX; Sigma‐Aldrich), 0.25 μM dexamethasone, 1 μg/mL insulin, 0.2 nM 3,3′,5-triiodo‑L‑thyronine (T3), and 1 μM pioglitazone. For WALC differentiation, the same protocol was followed, excluding pioglitazone.

MSCs cultured in basal medium were used as a negative control. Media were changed every 3 days. After 21 days, the differentiated cells were fixed and stained with 0.2% Oil Red O to visualize cytosolic LDs. Morphological changes were further confirmed through hematoxylin and eosin staining.

*UCP1* and *PLIN1* expression levels were analyzed via quantitative reverse transcription polymerase chain reaction (qRT‑PCR). RNA was extracted, reverse-transcribed, and quantified using SYBR Green (Ampliqon, Denmark) in a real‑time PCR system (Roche 9000, Switzerland). *GAPDH* was utilized as the reference gene, with relative gene expression calculated using 2^−ΔΔCt^. Gene-specific primers were designed using Oligo7 software (Molecular Biology Insights, CO), and their sequences are listed in S1 Table.

Western blot analysis was performed on undifferentiated MSCs, BALCs, and WALCs. Cells were lysed in RIPA buffer containing Tris-HCl, EDTA, NaCl, sodium deoxycholate, SDS, a protease inhibitor cocktail, and 1% Triton X‑100 (Thermo Fisher Scientific, MA). Protein concentrations were determined using a Bradford assay kit (VWR, PA). Equal amounts of protein were separated by SDS-PAGE and transferred onto PVDF membranes (Millipore, MA). The membranes were blocked with 2% non-fat dry milk in Tris-buffered saline with 0.1% Tween-20.

For protein detection, primary antibodies against UCP1 (Santa Cruz Biotechnology, TX), PLIN1 (Abcam, MA), and β-actin or GAPDH (Santa Cruz Biotechnology) were incubated overnight at 4°C. Next, HRP-conjugated secondary antibodies (Santa Cruz Biotechnology) were incubated for 90 min. Protein bands were visualized via enhanced chemiluminescence (ECL) (Thermo Fisher Scientific) and quantified with ImageJ (version 1.53c, NIH, MD), with normalization to β-actin or GAPDH.

### Preparation of adipocyte-free derivatives: conditioned media and exosomes

Once hADSC differentiation into BALCs and WALCs was confirmed, conditioned media (CM) were collected every 72 h and centrifuged to remove debris. Half of the CM was stored at −80°C (BALC-CM and WALC-CM). The remaining CM was subjected to exosome isolation using the AnaCell Exosome Kit (Ana Cell, Iran) according to our previously described protocol [24–26]. The filtered CM was mixed with reagent A (at a 5:1 ratio), incubated at 4°C for 12 h, and centrifuged. Supernatants were discarded, and exosome pellets (BALC-Exo and WALC-Exo) were resuspended in reagent B and stored at −80°C.

#### Characterization of adipocyte-derived exosomes.

The yield of the isolated exosomes was quantified through measuring the total protein content using a bicinchoninic acid (BCA) protein assay kit (Parstous, Iran), according to the manufacturer’s instructions. For subsequent functional assays, exosome preparations were normalized based on exosomal protein content. Exosome characterization included dynamic light scattering (DLS) using a Zetasizer (Malvern Instruments, UK) to measure the hydrodynamic radius, polydispersity index, and zeta potential. Field emission scanning electron microscopy (FE-SEM; MIRA3, TESCAN, Czech Republic) was performed on gold-sputtered samples. Transmission electron microscopy (TEM; EM-900, Carl Zeiss, Germany) was utilized to visualize exosomes on phosphotungstic acid-stained grids. The exosomal markers CD9, CD63, and TSG101 were detected via Western blotting. Calnexin was assessed as a negative control to verify the purity of the isolated exosomes.

### Cell selection and treatment

To explore the paracrine effects of CM and isolated exosomes derived from BALCs and WALCs, three cell types—primary hADSCs, OGCs, and hUCMSCs—and one established cell line, MCF‑7 breast cancer cells, were selected. hUCMSCs were isolated from full‑term normal deliveries and cultured according to our previously described protocol [27,28]. All treatments were carried out at an equal exosome protein concentration of 20 µg/mL.

#### Exosome uptake assay.

Exosome uptake was evaluated using the lipophilic membrane dye DiI (CellBrite™ Cytoplasmic Membrane Dye, Biotium) according to the manufacturer’s instructions. Briefly, isolated exosomes were labeled with DiI (5 µL/mL in PBS) and incubated for 20 min at 37 °C in the dark. The labeled exosomes were then added to hADSCs and incubated for 24 h. After incubation, the cells were washed with PBS in order to remove non‑internalized vesicles and analyzed by fluorescence microscopy (Olympus BX50, Olympus, Japan) to visualize as well as assess exosome internalization.

#### Isolation and culture of granulosa cells.

OGCs were isolated from a pool of follicular fluid obtained from IVF patients at the Alzahra Hospital Infertility Center, Tabriz, Iran. Oocyte donors under the age of 35 were chosen for the study. The remaining follicular fluids from oocyte retrieval were centrifuged at 1000 × g for 5 min. The pellet was resuspended in PBS and layered onto Ficoll-Paque reagent (Uppsala, Sweden; 17-0840-02), followed by centrifugation at 400 × g for 30 min. OGCs were collected from the grayish-white buffy coat layer, washed twice with PBS, and cultured in DMEM/F12 basal medium supplemented with 20% FBS. Cells from passages 2–3 were employed for subsequent experiments.

#### Characterization of isolated granulosa cells.

Isolated OGCs were characterized via flow cytometry based on the expression of CD105, CD44, and CD29, similar to the characterization of hADSCs. Granulosa cell markers FSHR and AMH (Santa Cruz Biotechnology) were analyzed through Western blot using HRP-conjugated antibodies. Immunocytochemistry was performed to confirm FSHR expression. OGCs were fixed with 4% paraformaldehyde, permeabilized with Triton X-100 (Sigma-Aldrich), blocked with 1% BSA, then incubated with an anti-FSHR antibody (Abcam, ab75200) followed by an FITC-conjugated secondary antibody (Elabscience, TX). The samples were visualized using an inverted fluorescence microscope (Olympus BX50, Japan).

#### Estradiol secretion (aromatase activity).

Aromatase, produced by OGCs, converts androgens into estrogens [19]. To assess aromatase activity, OGCs were cultured and treated with conditioned media as well as exosomes for 48 h. Testosterone was added during the final 24 h. Estradiol (E_2_) levels were measured using a Cobas e411 analyzer (Hitachi/Roche) equipped with second-generation electrochemiluminescence technology.

#### Apoptosis and expression of apoptosis-associated genes.

MCF-7 cells were treated with conditioned media and exosomes for 48 h. Apoptosis profiles were evaluated using Annexin V-FITC staining (eBioscience™, CA) followed by flow cytometry analysis. Data were processed using CellQuest software.

In parallel, the expression of apoptosis‑related genes (*Bcl-2*, *Bax*, and *Caspase-3*) was analyzed via reverse transcription followed by quantitative PCR (qPCR). Primer sequences used in this study are outlined in S1 Table.

Evaluation of osteogenic differentiation: ALP activity, Alizarin Red S staining, and expression of osteogenic markers

For evaluating alkaline phosphatase (ALP) activity, hADSCs were treated with conditioned media and exosomes in osteogenic media for 7, 14, and 21 days. The cells were fixed with 10% formalin, with ALP staining performed using a commercial kit (Asiapajohesh, Iran). Quantitative analysis was performed through measuring the optical density at 405 nm using a microplate reader (Stat Fax 4200, Awareness Technologies, FL).

To appraise the effects of conditioned media and exosomes on calcium deposition in hADSCs, Alizarin Red S staining was performed on days 7, 14, and 21. For this purpose, cells were cultured in 24-well plates and washed with PBS. The cells were then fixed with 10% formalin at 4°C for 1 h and stained with 2% Alizarin Red S solution (pH 4.2) for 30 min, followed by three washes to remove excess stain. The bound dye was extracted with 10% acetic acid, neutralized, and quantified spectrophotometrically (typically 405 nm).

To evaluate the expression of osteogenic genes, hADSCs were cultured in osteogenic induction medium supplemented with conditioned media and exosomes. Total RNA was extracted from samples at the same time points (days 7, 14, and 21). The expression levels of key osteogenic marker genes—specifically *Runx2*, *Osteopontin* (OP), and *Osteocalcin* (OC)—were analyzed via qRT-PCR. Primer sequences for these target genes are provided in S1 Table.

#### Stemness features in somatic, stem, and breast cancer cells.

The paracrine effects of BALCs and WALCs on stemness were evaluated in the cells. Primary antibodies against Sox2 (Santa Cruz Biotechnology) and Oct4 (Santa Cruz Biotechnology) were used.

### Lipid profiling

Cells were treated with exosomes for 72 h. Cell pellets were collected, dried, and resuspended in water. Lipids were extracted using methanol, methyl tert-butyl ether, and chloroform (1.33:1:1) with butylated hydroxytoluene as an antioxidant (Sigma-Aldrich, Germany) plus a SPLASH internal standard (Avanti Polar Lipids, AL), following a modified Pellegrino et al. protocol [29,30]. Samples were vortexed, incubated, and centrifuged. The supernatants were collected and dried, and the remaining supernatants were pooled for quality control. The dried samples were resuspended, centrifuged, and transferred to LC-MS vials.

LC-MS analysis was performed on a Q-Exactive Orbitrap (Thermo Fisher Scientific) coupled to a Dionex Ultimate 3000 LC system. An Accucore C30 RP column was used with a gradient of AcN/H_2_O/FA/ammonium acetate (Solvent A) and IPA/AcN/FA/ammonium acetate (Solvent B) at a flow rate of 0.26 ml/min. The solvents were LC-MS grade (Merck). The gradient ranged from 30% to 100% B over 20 min, followed by a wash step and re-equilibration [29,31].

LC-MS/MS data were acquired in positive and negative ion modes (70,000/17,500 resolution) using data-dependent acquisition [29,32]. Data analysis was carried out using Compound Discoverer 3.3 (Thermo Fisher Scientific, Germany). Lipid species were identified based on mass, retention time, fragmentation, and isotopic pattern using in silico databases [33].

Lipid species are represented either as sum composition (carbons and double bonds) or, if possible/unambiguous with the composition of acyl chains. All lipidomics values represent relative peak areas; the raw data (area under the peak) were normalized to the corresponding internal standard.

### Statistical analysis

Cell culture experiments were performed in triplicate unless otherwise specified. Data are presented as mean ± standard deviation (SD) and were analyzed using one-way analysis of variance (ANOVA), followed by Tukey’s post hoc test (GraphPad Software, CA, USA). A p-value < 0.05 was considered statistically significant.

## Results

### Phenotype and plasticity of hADSCs

**Fig 2:**
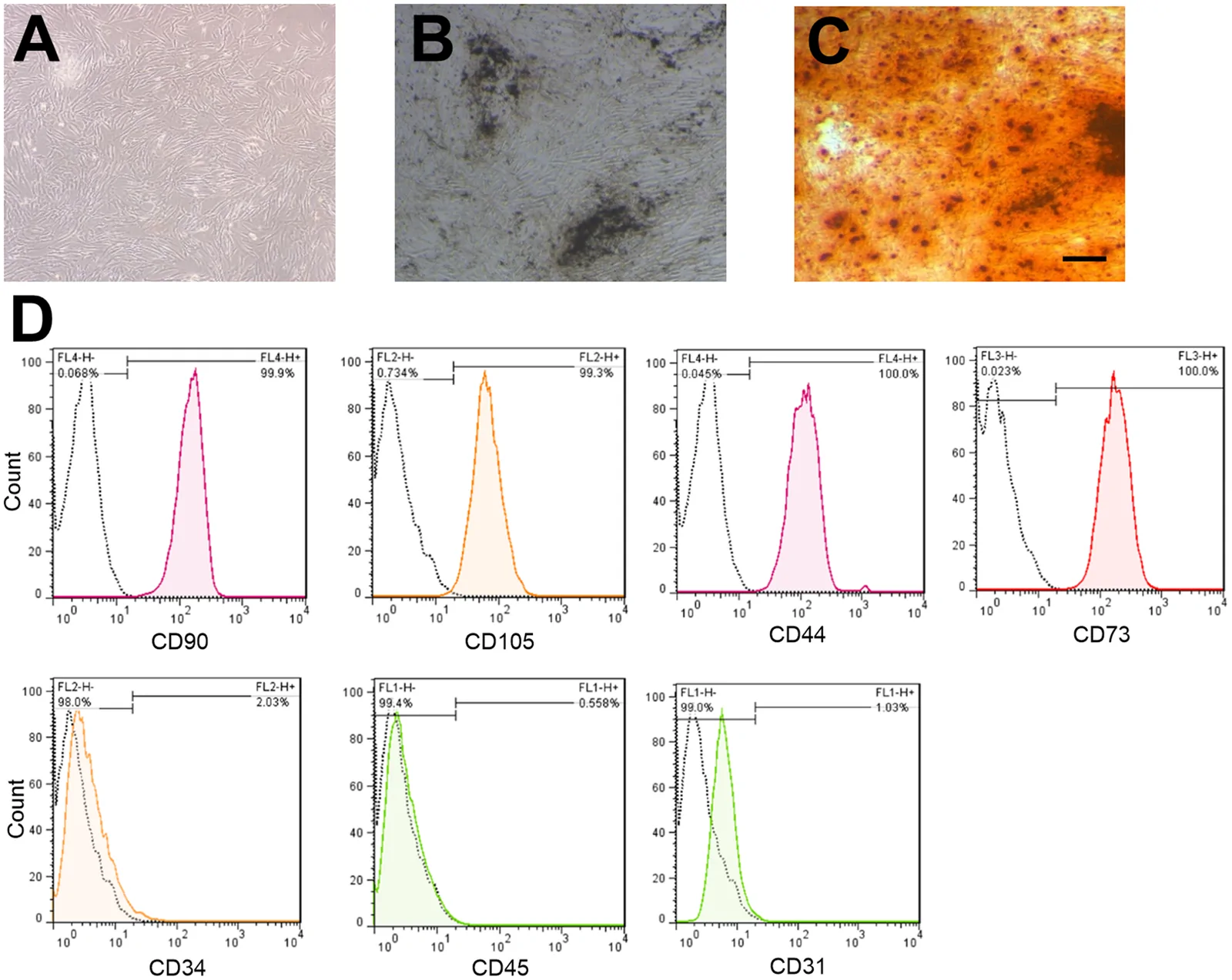
Characterization of isolated mesenchymal stem cells (MSCs). Representative brightfield image of MSCs exhibiting a spindle-shaped, fibroblast-like morphology in culture (**A**). Osteogenic differentiation of MSCs visualized before (**B**) and after (**C**) Alizarin Red S staining of calcium deposits (scale bar = 100 µm). Flow cytometry analysis of MSC surface markers (**D**). Histograms demonstrate high expression of positive markers (CD90 (99.9%), CD105 (99.3%), CD44 (100%), CD73 (100%)) and low or negative expression of hematopoietic as well as endothelial markers (CD34 (2.03%), CD45 (0.558%), CD31 (1.03%)), confirming the MSC phenotype.

**Fig 3:**
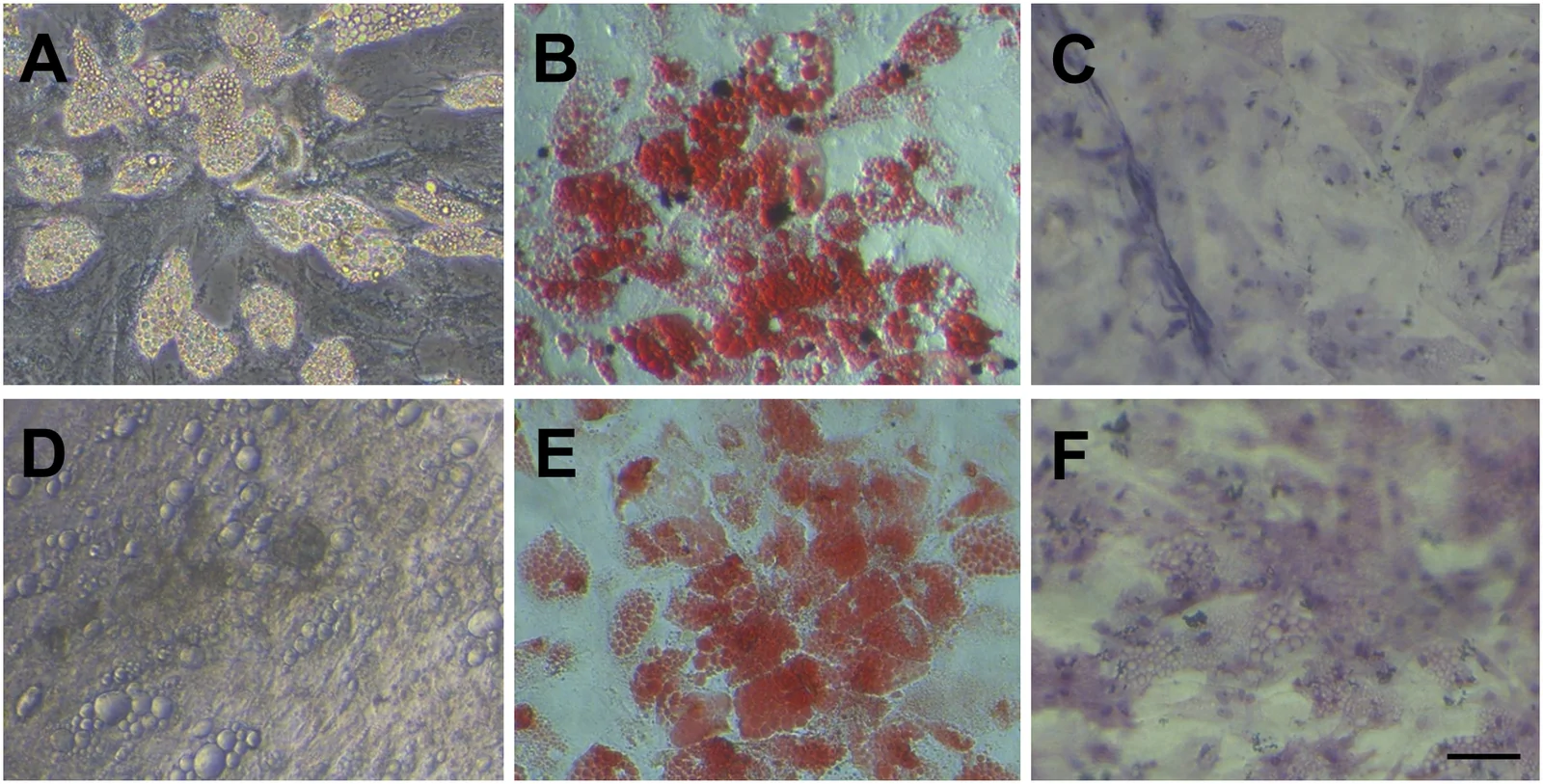
Adipogenic differentiation into brown adipocyte-like cells (BALCs) and white adipocyte-like cells (WALCs). Oil Red O staining of BALCs before (**A**) and after (**B**) staining, and WALCs before (**D**) and after (**E**) staining. Hematoxylin and Eosin (H&E) staining reveals larger lipid droplets in the cytoplasmic vacuoles of WALCs (**F**) compared to BALCs (**C**) (scale bar = 20 μm).

The hADSCs presented uniform adherence and spindle-like morphology (Fig 2A). Alizarin Red S staining confirmed osteogenic differentiation after 21 days (Fig 2B and 2C). Flow cytometry revealed that hADSCs expressed MSC markers (≥99%), including CD90, CD105, CD44, and CD73 and were negative or expressed very low levels of CD34, CD45, and CD31 (≤2.5%) (Fig 2D). Adipogenic differentiation revealed LDs in differentiated adipocytes. BALCs contained smaller LDs than WALCs (Fig 3A and 3D), as confirmed by Oil Red O and H&E staining (Fig 3B, 3E, 3C, and 3F). These findings confirm the successful isolation of multipotent hADSCs.

### Changes in adipogenic markers: UCP1 and PLIN1

**Fig 4:**
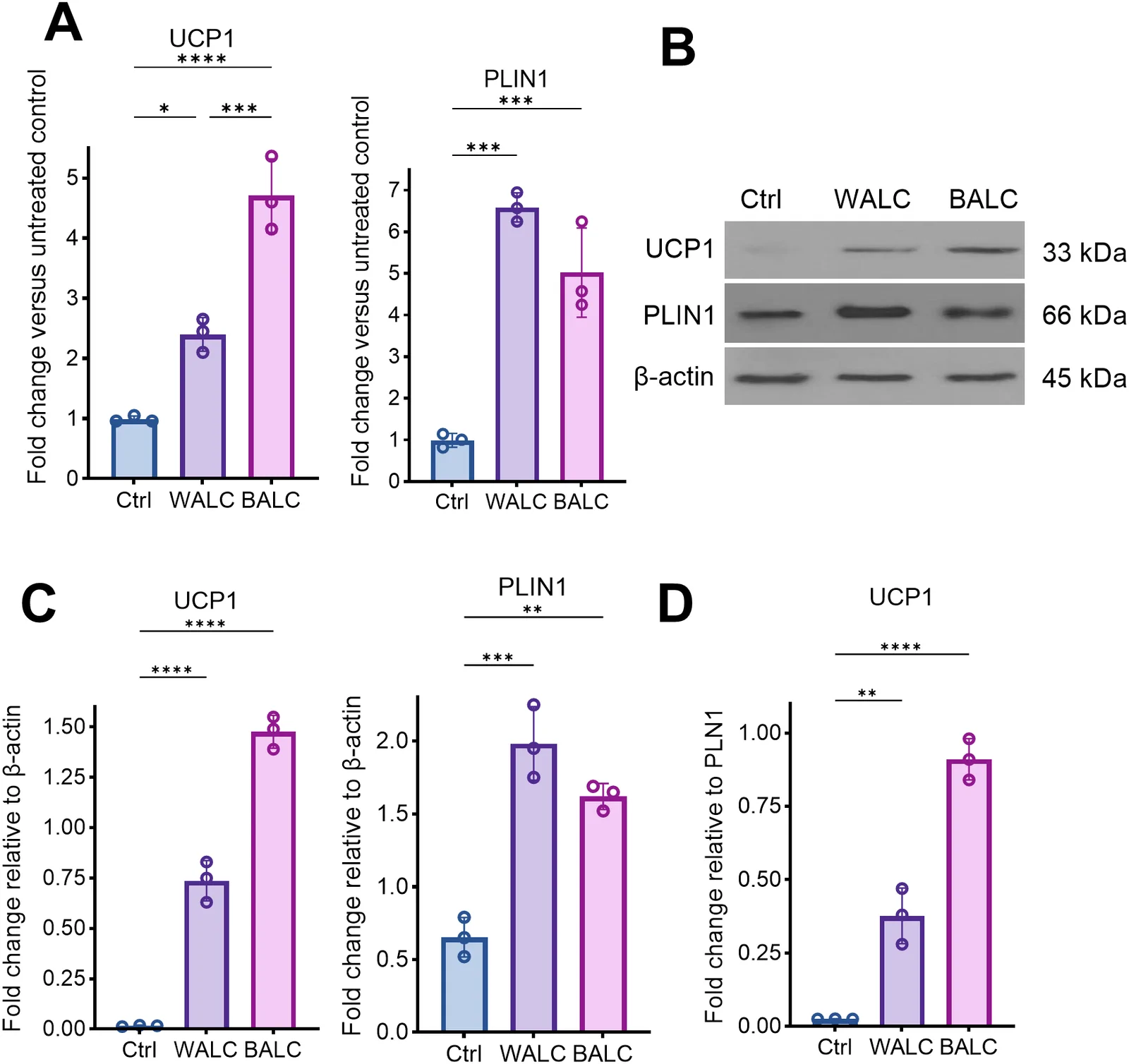
Gene expression levels of UCP1 and PLIN1 were quantified in white adipocyte-like cells (WALCs) and brown adipocyte-like cells (BALCs) relative to the untreated control (A). Data are presented as fold change versus the untreated control. Protein expression levels of UCP1, PLIN1, and β‑actin (loading control) were assessed by Western blot analysis (**B**). UCP1 and PLIN1 protein levels were quantified as fold change relative to β‑actin (**C**). UCP1 protein expression was normalized to PLIN1 (**D**). Asterisks denote statistical significance: *p < 0.05, **p < 0.01, ***p < 0.001, ****p < 0.0001.

Gene expression analysis confirmed the differentiation of hADSCs into BALCs and WALCs. The results revealed a significantly high expression of the UCP1 marker in BALCs (2.0-fold versus WALCs, p < 0.001). In comparison with undifferentiated hADSCs, the expression of the PLIN1 marker was higher in both WALCs (6.6-fold, p < 0.001) and BALCs (5.1-fold, p < 0.001; Fig 4A and 4B).

The UCP1 protein expression in BALCs was also higher than that in WALCs and undifferentiated MSCs (2.0-fold, p < 0.0001). Compared with the control group, PLIN1 protein expression was augmented in both BALCs (2.5-fold, p < 0.01) and WALCs (3.0-fold, p < 0.001) (Fig 4C). The UCP1/PLIN1 protein expression ratio in BALCs was 2.5-fold higher than that in WALCs (Fig 4D).

### Characterization of adipocyte-derived exosomes

**Fig 5:**
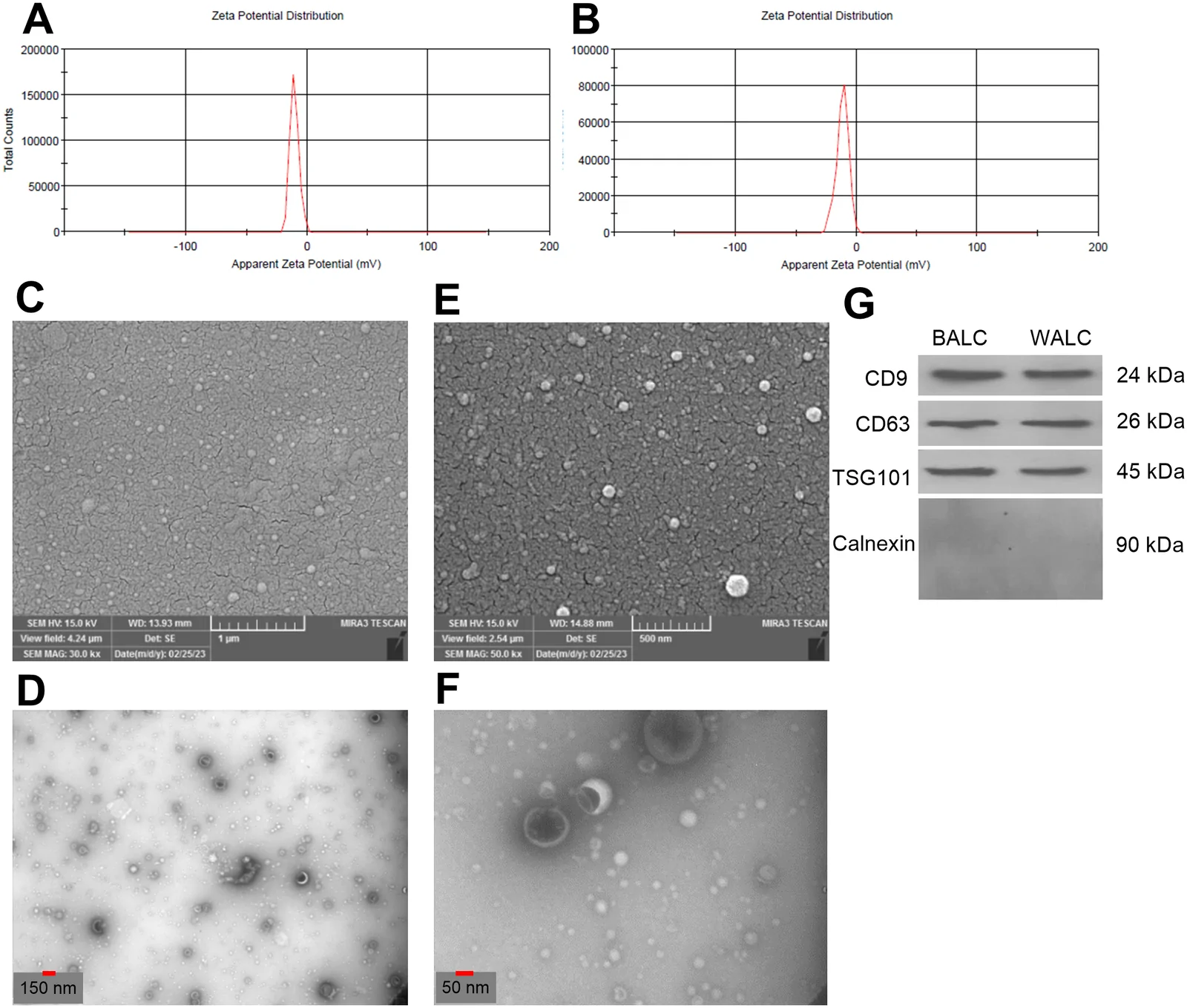
Characterization of exosomes derived from adipocyte-like cells. Zeta potential distribution of exosomes isolated from brown adipocyte-like cells (BALCs) (**A**) and white adipocyte-like cells (WALCs) (**B**) conditioned media. Scanning electron microscopy (SEM) and transmission electron microscopy (TEM) images indicating the morphology of BALC (**C, D**) and WALC (**E, F**) -derived exosomes at specified magnifications. Western blot analysis of typical exosomal markers (CD9, CD63, and TSG101) and the endoplasmic reticulum protein Calnexin, as a negative marker, in exosomes isolated from BALCs and WALCs (**G**).

The isolated exosomes were characterized according to standard criteria, including protein content assay, particle size distribution analysis, surface charge measurement, morphological assessment, and detection of exosomal markers. The yield of exosomes was determined through quantifying the protein content of each sample using the BCA protein assay. Standard curve of the BCA assay is displayed in S1 Fig. The average size of the exosomes was approximately 60 nm. Zeta potential analysis revealed that BALC-derived exosomes had a zeta potential of −10.4 mV, while WALC-derived exosomes had a zeta potential of −11.4 mV (Fig 5A and 5B). FE-SEM results demonstrated that the size distribution of exosomes from both BALCs and WALCs was within the typical exosome size range of approximately 30–150 nm (Fig 5C and 5E). TEM imaging further confirmed the morphology of the exosomes, which appeared nearly spherical and consistent in size (Fig 5D and 5F). Western blotting analysis verified that exosomes derived from BALC and WALC express typical exosomal markers (CD9, CD63, and TSG101), but do not express the endoplasmic reticulum marker (Calnexin) (Fig 5G). These findings collectively confirmed the successful isolation of BALC‑ and WALC‑derived exosomes.

### In vitro tracking of fluorescently labeled exosomes

**Fig 6:**
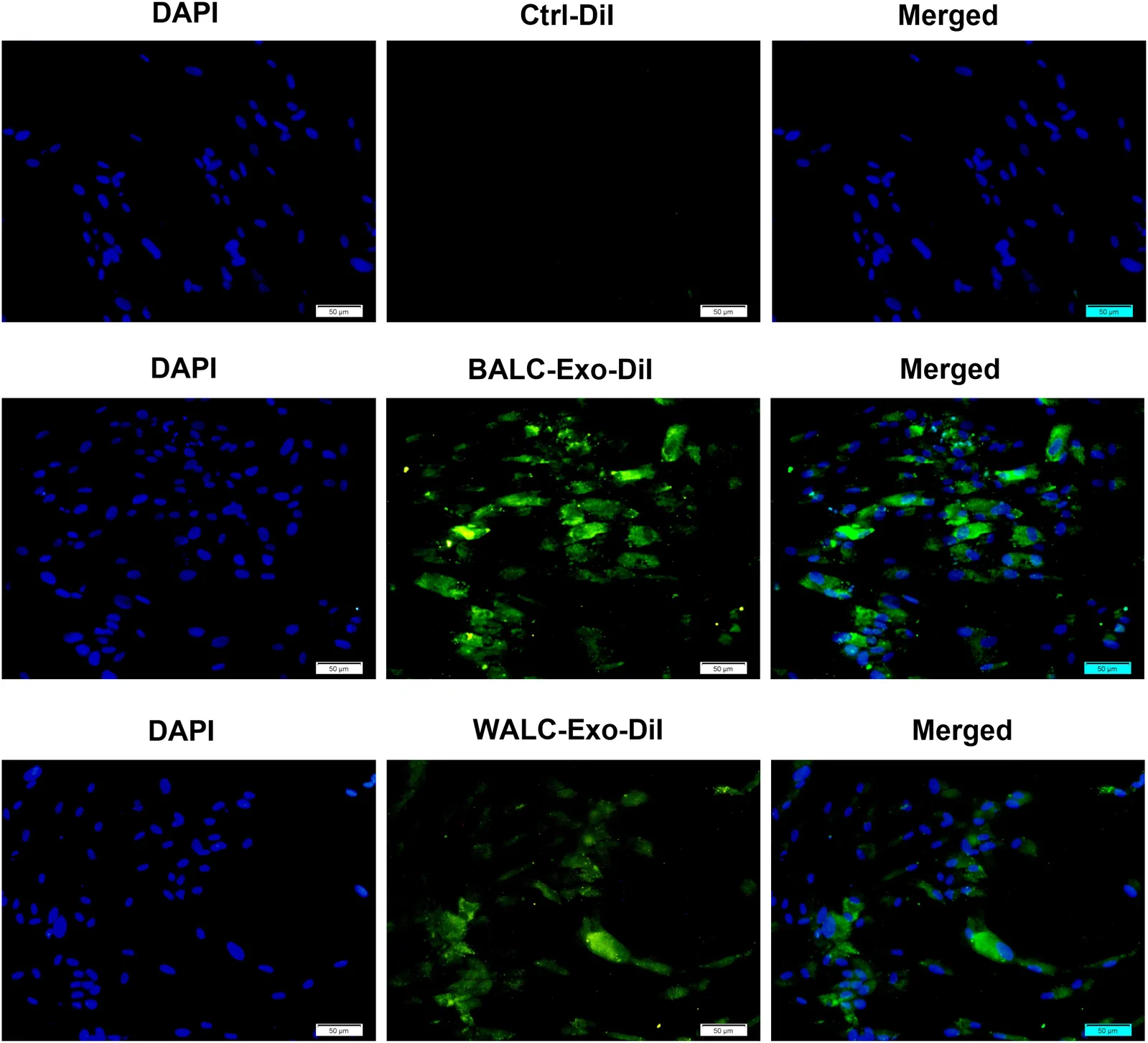
*In vitro* tracking of exosomes. Cellular uptake of exosomes derived from brown and white adipocyte-like cells (BALC-Exo and WALC-Exo) was evaluated using fluorescent membrane labeling. DiI-labeled BALC-Exosome and WALC-Exosome (green) were treated with hADSCs. After 24 h of treatment, cell nuclei were stained with DAPI (blue) and visualized by fluorescence microscopy. Scale bar = 50 μm.

Fluorescence microscopy was used to monitor the uptake of DiI‑labeled exosomes by hADSCs. Briefly, exosomes were labeled with the lipophilic fluorescent dye DiI and incubated with hADSCs for 24 h. Fluorescence imaging exhibited successful internalization of the labeled exosomes to the cells (Fig 6).

### Isolation and characterization of human ovarian granulosa cells

**Fig 7:**
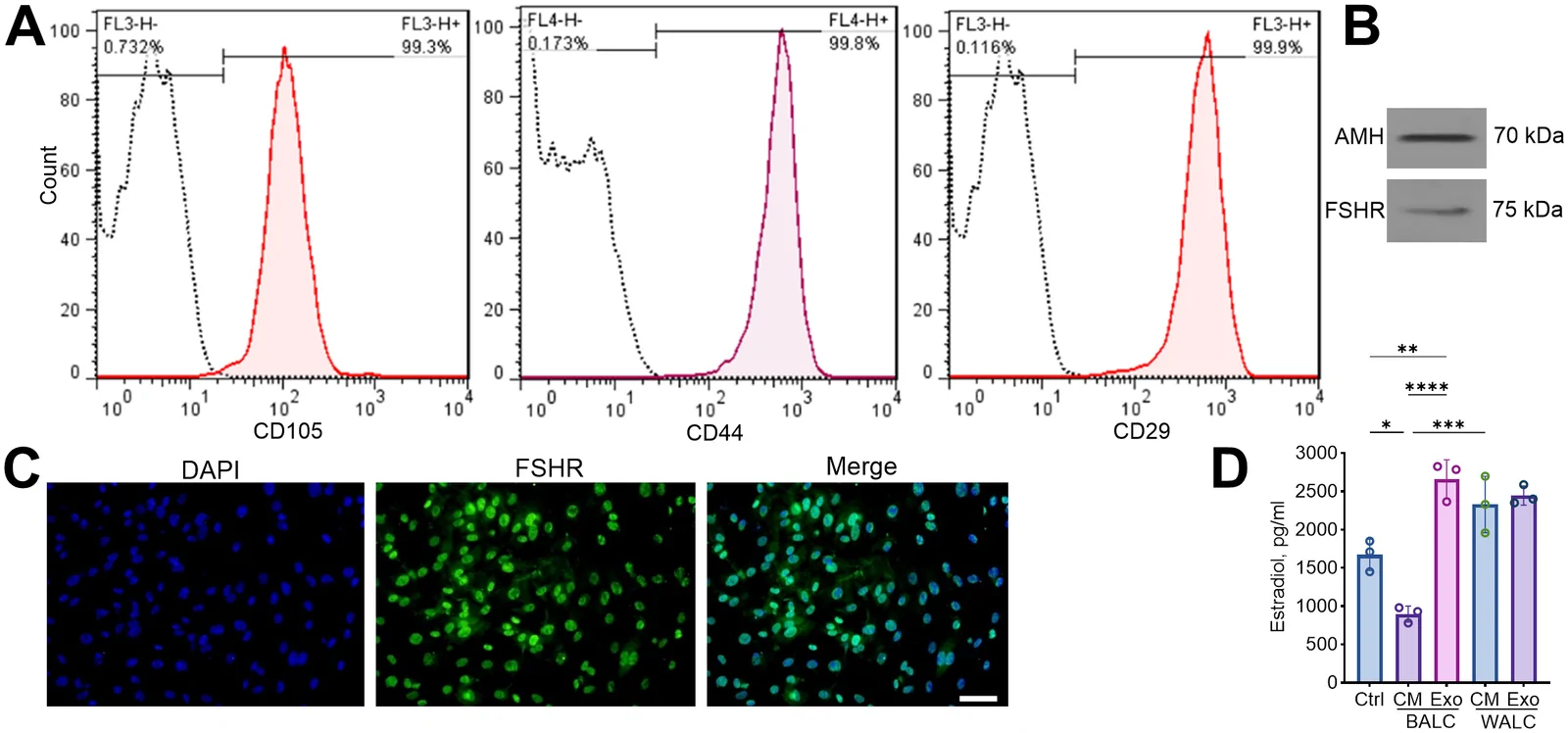
Characterization of ovarian granulosa cells. Flow cytometry analysis indicating the expression of surface markers in ovarian granulosa cells (OGCs). Dotted lines represent isotype controls, and filled histograms represent specific antibody staining for CD105 (PerCP-Cy5.5), CD44 (APC), and CD29 (PE-Cy7) (**A**). Western blot analysis of AMH and FSHR (**B**). Molecular weights are indicated on the right. Immunofluorescence staining of OGCs (**C**). Cells were stained with DAPI (blue) to visualize nuclei and with an antibody against FSHR (green) to present its expression. The scale bar represents 50 µm. Estradiol secretion levels in conditioned media from control (Ctrl) cells, conditioned media from brown adipocyte-like cells (BALCs-CM), exosomes from brown adipocyte-like cells (BALCs-Exo), conditioned media from white adipocyte-like cells (WALCs-CM), and exosomes from white adipocyte-like cells (WALCs-Exo) (**D**). Asterisks indicate statistical significance: *p < 0.05, **p < 0.01, ***p < 0.001, ****p < 0.0001.

The morphology of primary OGCs is shown in S2 Fig. The characterization of isolated OGCs was confirmed through the expression of cell surface markers, including CD105 (99.3%), CD44 (99.8%), and CD29 (99.9%), as assessed by FACS analysis (Fig 7A). Further, the expression of FSHR and AMH, which are commonly used as key biomarkers of OGCs, was verified by Western blotting (Fig 7B). Ultimately, immunofluorescence staining of OGCs (Fig 7C) verified their presence in the experimental setup.

#### Differential effects on estradiol secretion.

To evaluate the impact of the treatments on functional capacity, OGCs were exposed to CM and exosomes. Compared with the control group, treatment with BALCs-CM significantly lowered estradiol concentration, indicating approximately a 2.5-fold reduction. In contrast, treatment with BALC-derived exosomes led to a 1.5-fold growth in estradiol concentration. Likewise, treatment with WALCs‑CM and WALC‑derived exosomes resulted in elevated estradiol concentration, with approximately 1.2-fold and 1.5-fold changes, respectively, in the culture medium (Fig 7D).

### Altered apoptosis and apoptosis markers

**Fig 8:**
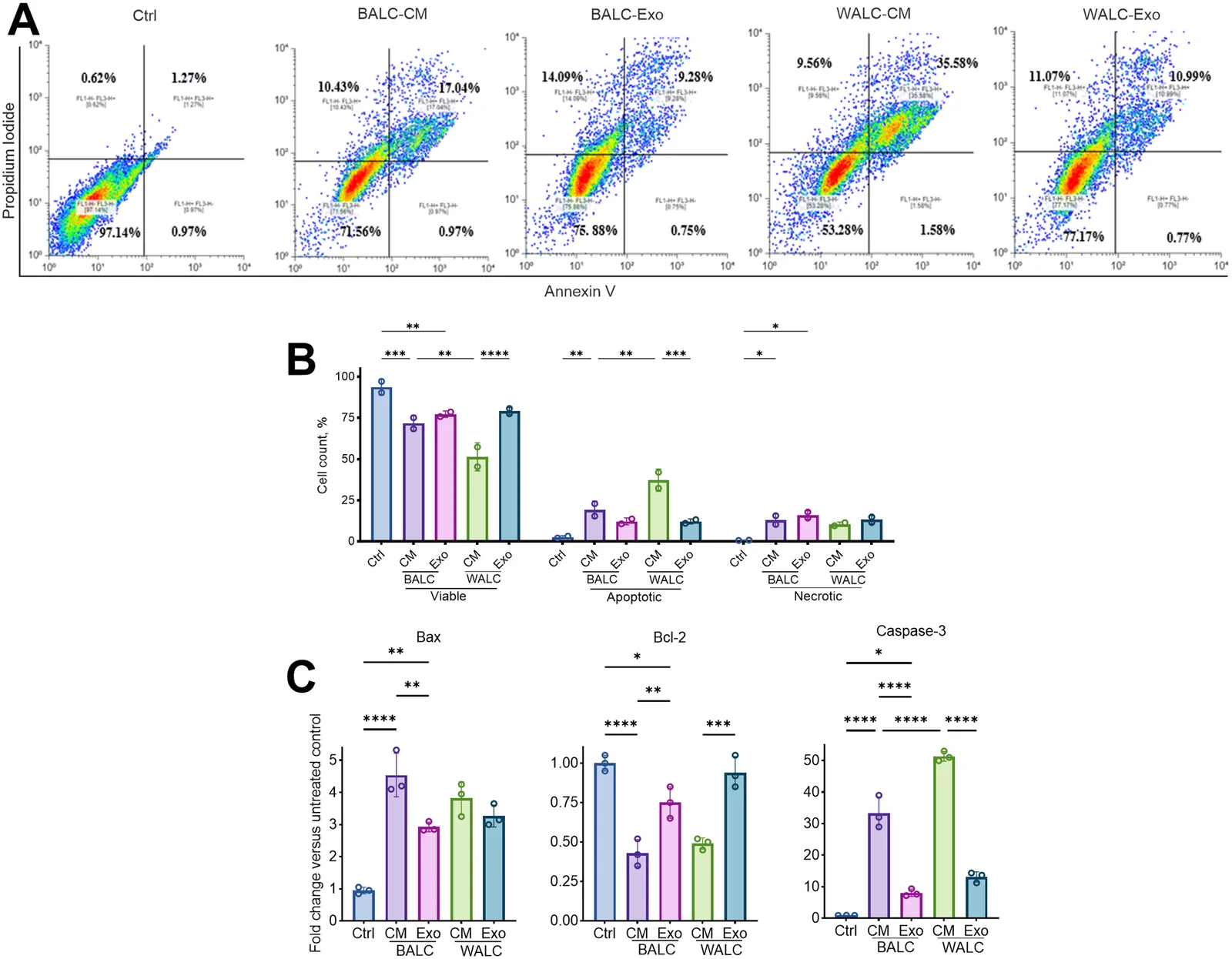
Adipocyte-derived modulation of MCF-7 apoptosis. Representative flow cytometry plots revealing the distribution of viable, early apoptotic, and late apoptotic/necrotic cells (**A**). Cells were treated with control (Ctrl) media, conditioned media (CM) or exosomes from brown adipocyte-like cells (BALCs) or white adipocyte-like cells (WALCs) for 48 h. Cells were stained with Annexin V and propidium iodide (PI) to distinguish cell populations. Quantification of viable, apoptotic, and necrotic cell populations from flow cytometry analysis (**B**). Data are expressed as the percentage of total cells. Gene expression levels of pro-apoptotic (*Bax, Caspase-3*) and anti-apoptotic (*Bcl-2*) markers were quantified by qRT‑PCR in cells treated with conditioned media or exosomes derived from BALCs and WALCs for 24 h (**C**). Asterisks denote statistical significance: *p < 0.05, **p < 0.01, ***p < 0.001, ****p < 0.0001.

In the cancer cell model, all treatments significantly reduced cell viability and increased apoptosis compared with the control group, with WALCs-CM demonstrating the strongest apoptotic effect (Fig 8A and 8B). WALC-derived exosomes exerted a smaller decline in cell viability compared to WALC-CM. Notably, only BALC-derived factors significantly promoted necrosis.

Pro-apoptotic *Bax* mRNA was upregulated in all treated groups (p < 0.0001, p < 0.001), leading to a lowered *Bcl2*/*Bax* ratio. BALCs-CM and WALCs-CM significantly downregulated anti-apoptotic *Bcl2* mRNA expression, while BALC-derived exosomes relatively diminished its expression (p < 0.05), and WALC-derived exosomes showed no significant change (Fig 8C). BALCs-CM and WALCs-CM significantly upregulated *Caspase*-3 expression (p < 0.0001), reflecting activation of apoptosis via the Bcl2/Bax and Caspase-3 pathways. These results suggest the potential of WALCs-CM and BALCs-CM in promoting breast cancer cell apoptosis.

### Osteogenic differentiation potential

**Fig 9:**
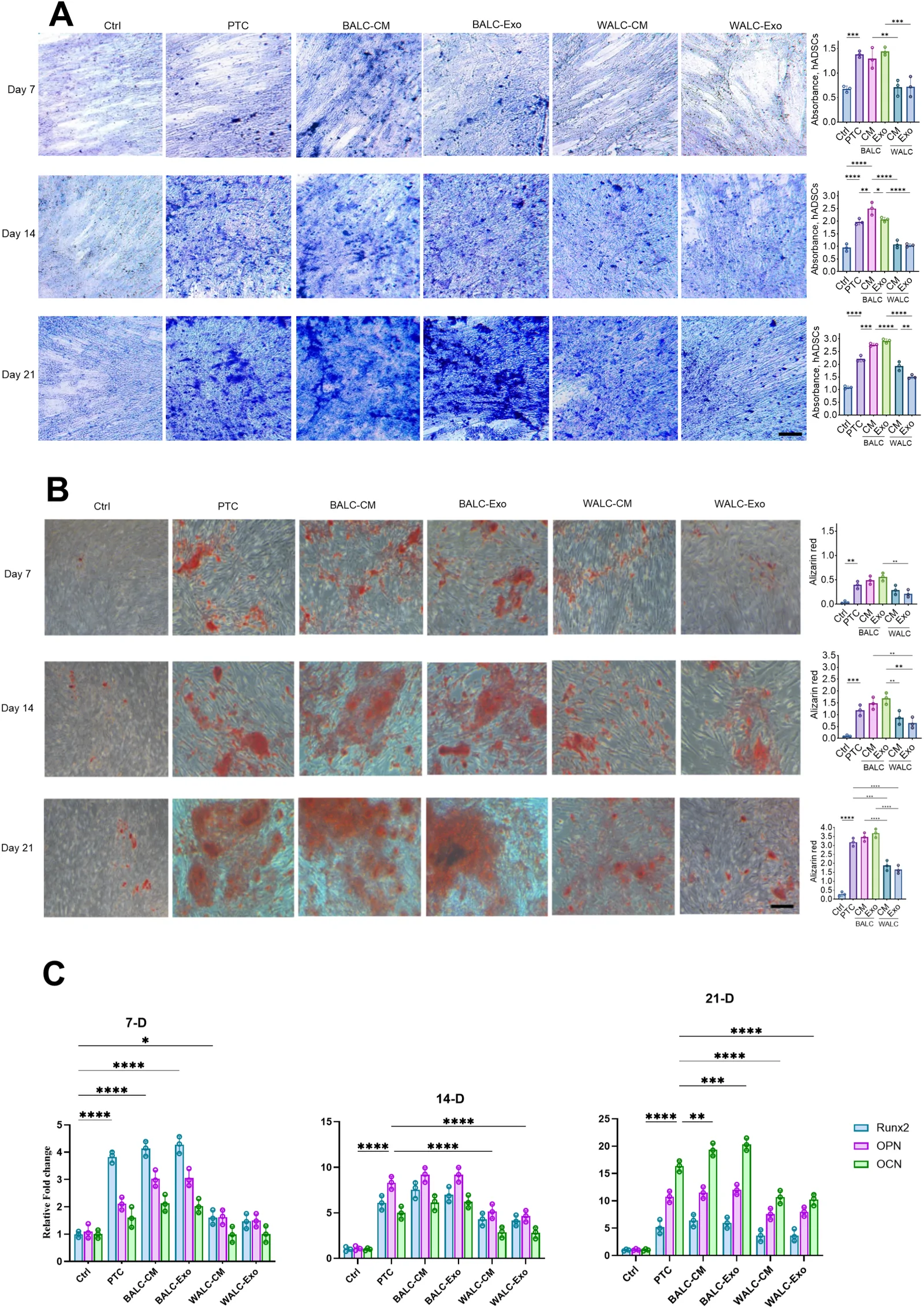
Effects of various treatments on the osteogenic differentiation of cultured human adipose-derived stem cells (hADSCs) were assessed at different time points. **(A)** Osteogenic capacity was evaluated through a semi-quantitative analysis of alkaline phosphatase (ALP) activity and spectrophotometric measurement of ALP activity on days 7, 14, and 21. (**B**) Alizarin Red S staining and quantification of osteogenic differentiation in hADSCs on days 7, 14, and 21. Representative images of Alizarin Red S staining showing increased mineralized nodule formation in hADSCs treated with BALC-CM and BALC-Exo compared to controls. (**C**) Gene expression levels of Runx2, osteopontin (OP), and osteocalcin (OC) were quantified by qRT-PCR in hADSCs treated with conditioned media and exosomes derived from BALCs and WALCs on days 7, 14, and 21. Ctrl (negative control), PTC (positive control), CM (conditioned medium), and Exo (exosome). Scale bars represent 10 μm and 100 μm. Asterisks represent statistical significance: *p < 0.05, **p < 0.01, ***p < 0.001, and ****p < 0.0001.

BALC-CM and BALC-derived exosomes treatments showed similar ALP activity, a marker for osteogenic differentiation, to positive control on day 7 (Fig 9A). By day 21, BALC-derived exosomes further enhanced ALP activity, exceeding the impact observed with BALC-CM (1.3- and 1.4-fold) (Fig 9A). To explore the effects of conditioned media and exosomes on the osteogenic differentiation of hADSCs, Alizarin Red S staining was carried out on days 7, 14, and 21 (Fig 9B). Interestingly, at all these time points, hADSCs treated with conditioned media and exosomes derived from BALCs exhibited significantly higher calcification compared to the positive control group (Fig 9B). These observations suggest that BALC-CM and BALC-Exo treatments enhance Ca^2+^ deposition more effectively than WALC-CM and WALC-Exo treatments.

qRT-PCR was applied on days 7, 14, and 21 to assess the expression of Runx2, osteopontin (OP), and osteocalcin (OC) (Fig 9C). The expression of Runx2 rose on day 7 in the BALC-CM and BALC-Exo groups compared to the positive control group. Further, comparison between the BALC-CM and BALC-Exo groups demonstrated a statistically significant difference in Runx2 expression, with BALC-Exo exerting a greater stimulatory effect on Runx2 upregulation. Next, OP expression increased on day 14 in the BALC-CM and BALC-Exo groups. On day 21, OC expression revealed a marked growth in the BALC-CM and BALC-Exo groups compared to the positive control group. Overall, BALC-CM and BALC-Exo treatments were more effective than WALC-CM and WALC-Exo treatments in elevating the expression levels of Runx2, osteopontin, and osteocalcin.

### Changes in stemness markers

**Fig 10:**
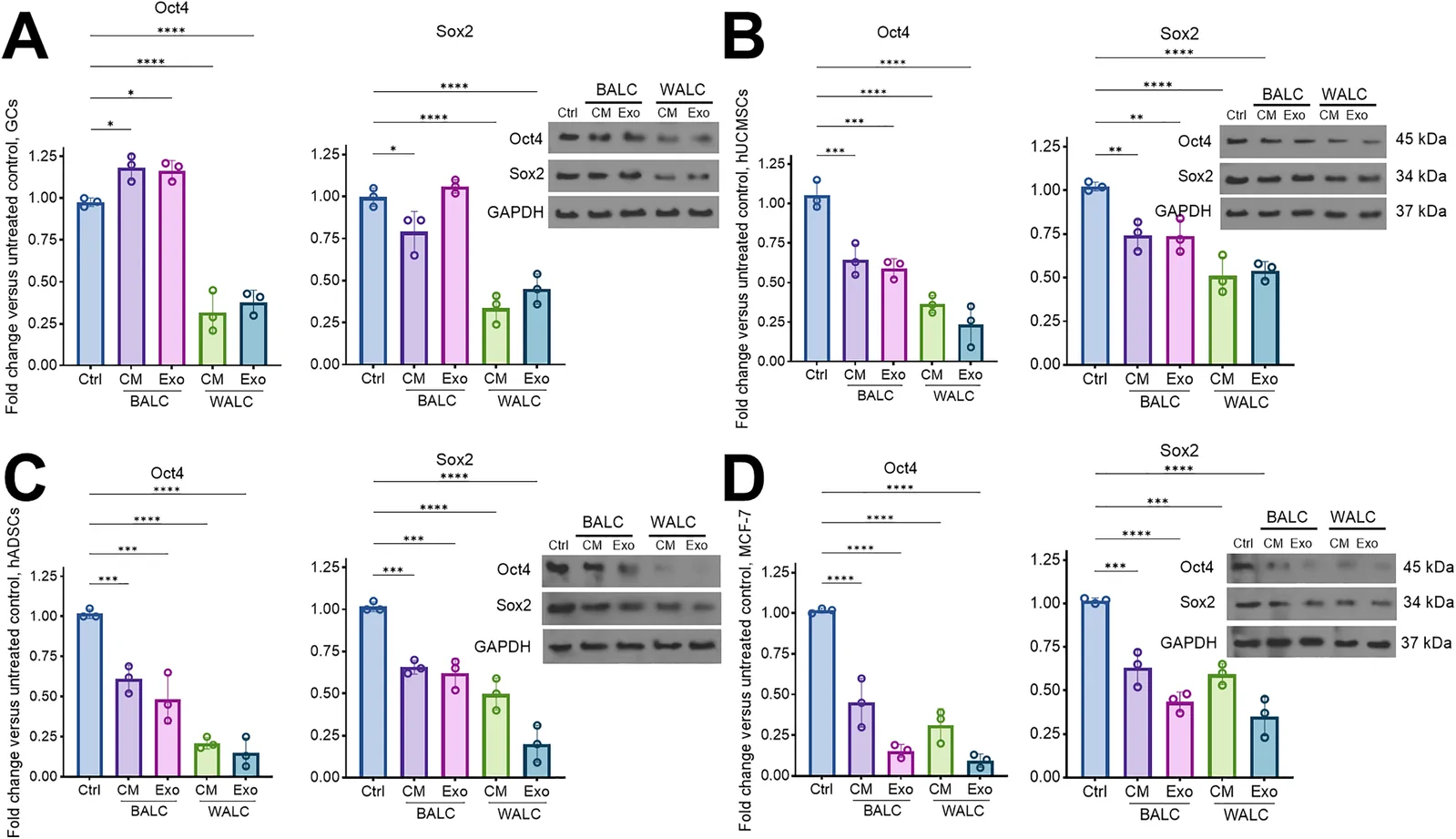
Stemness property changes in different cell types. Western blot analysis was performed to evaluate Sox2 and Oct4 protein levels, markers of stemness, in ovarian granulosa cells (OGCs) (**A**), human umbilical cord mesenchymal stem cells (hUCMSCs) (**B**), human adipose-derived stem cells (hADSCs) (**C**), and MCF-7 breast cancer cells (**D**). GAPDH served as the internal control. Asterisks reflect statistical significance: *p < 0.05, **p < 0.01, ***p < 0.001, ****p < 0.0001.

Oct4 and Sox2 are key genes involved in self-renewal. Compared with factors derived from WALCs, interactions between OGCs and paracrine factors from BALCs, especially BALC-derived exosomes, appeared to maintain or enhance stemness (Fig 10A), a feature essential for fertility and embryonic development. In other cell types, however, treatment with paracrine factors led to diminished stemness (Fig 10B–D).

Nevertheless, in hUCMSCs and hADSCs, the stemness markers were higher in treatments with BALC versus WALC-paracrine factors. Notably, in hUCMSCs and hADSCs, stemness marker expression remained higher following treatment with BALC-derived compared with WALC-derived paracrine factors. Interestingly, in MCF-7 breast cancer cells, exosomes suppressed stemness markers relative to CM, regardless of the adipocyte source.

### Changes in cellular lipidomic profile

**Fig 11:**
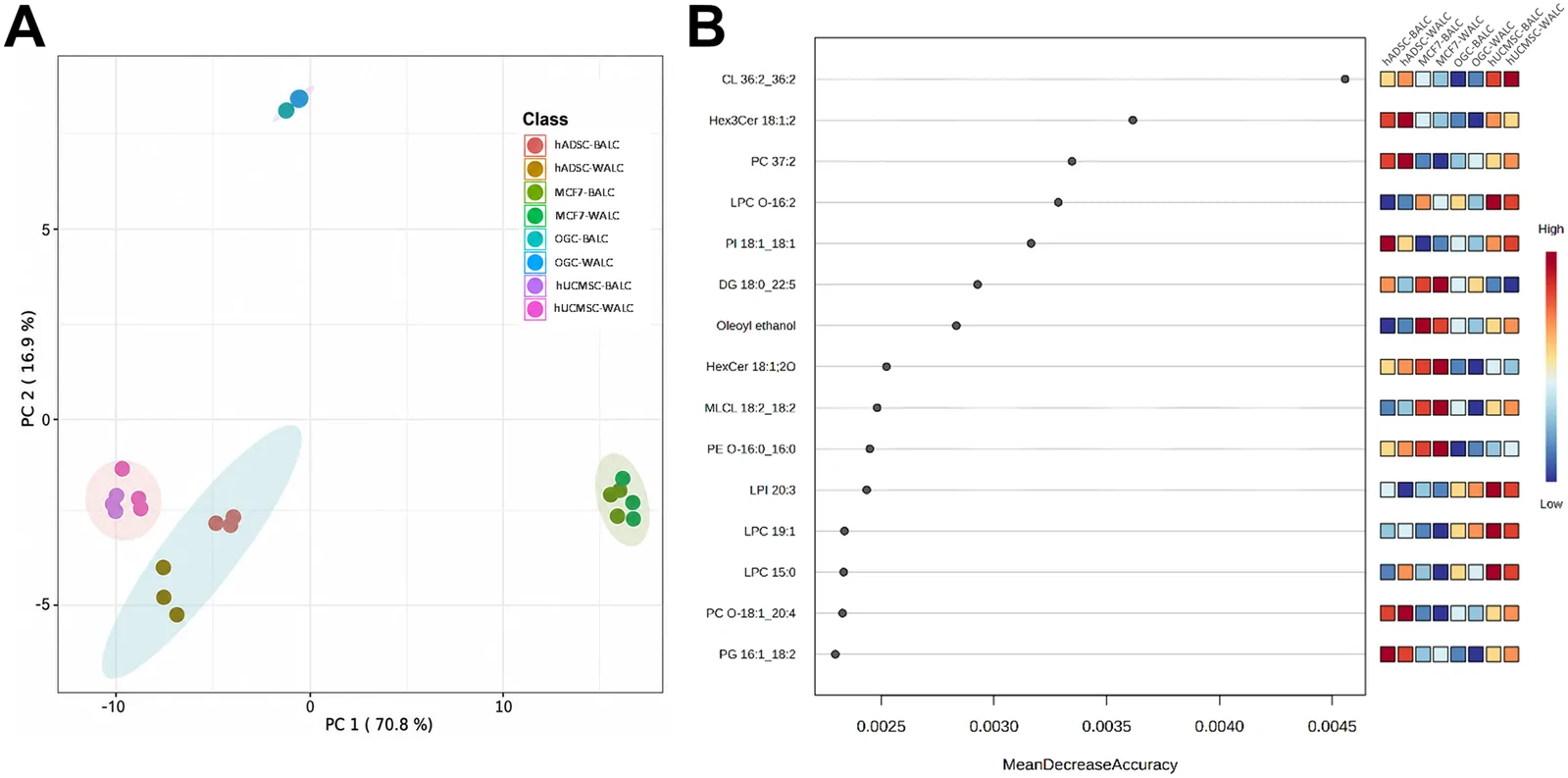
Differentiating cellular phenotypes based on lipidomic analysis. (**A**) Overview of the PCA score plot of all four cell lines in a 2D space. The clusters are reasonably well separated in different spaces of the PCA plot, revealing apparent differences between the cell lines. Sample normalization: by sum; Data transformation: Log transformation. (**B**) Feature importance plot generated from a Random Forest model.

LC-MS/MS identified 699 lipid species from 36 classes (S2 Table). The principal component analysis (PCA) score plot suggests that the cell lines can be grouped into distinct clusters based on their overall lipid similarity (based on K-means Clustering). The separation between the clusters in different spaces suggests that there are considerable differences between the cell lines (Fig 11A). The cluster of hADSCs treated with exosomes is larger and more dispersed (heterogenous) than the other clusters, presenting a clear separation between the BALC‑derived exosomes and WALC‑derived exosomes (Fig 11A).

The feature importance plot suggests that the lipids CL36:2, HexCer 18:1:2, and PC 0–16:2 indicate the most variation in lipid levels across different experimental groups or conditions. This signifies that removing these features would exert the most significant negative impact on the model's accuracy. In Fig 11B, the heatmap on the right reveals significant differences in the levels of these lipids across the different samples or groups. For instance, CL36:2 appears to be higher in the WALC group compared with the BALC group.

**Fig 12:**
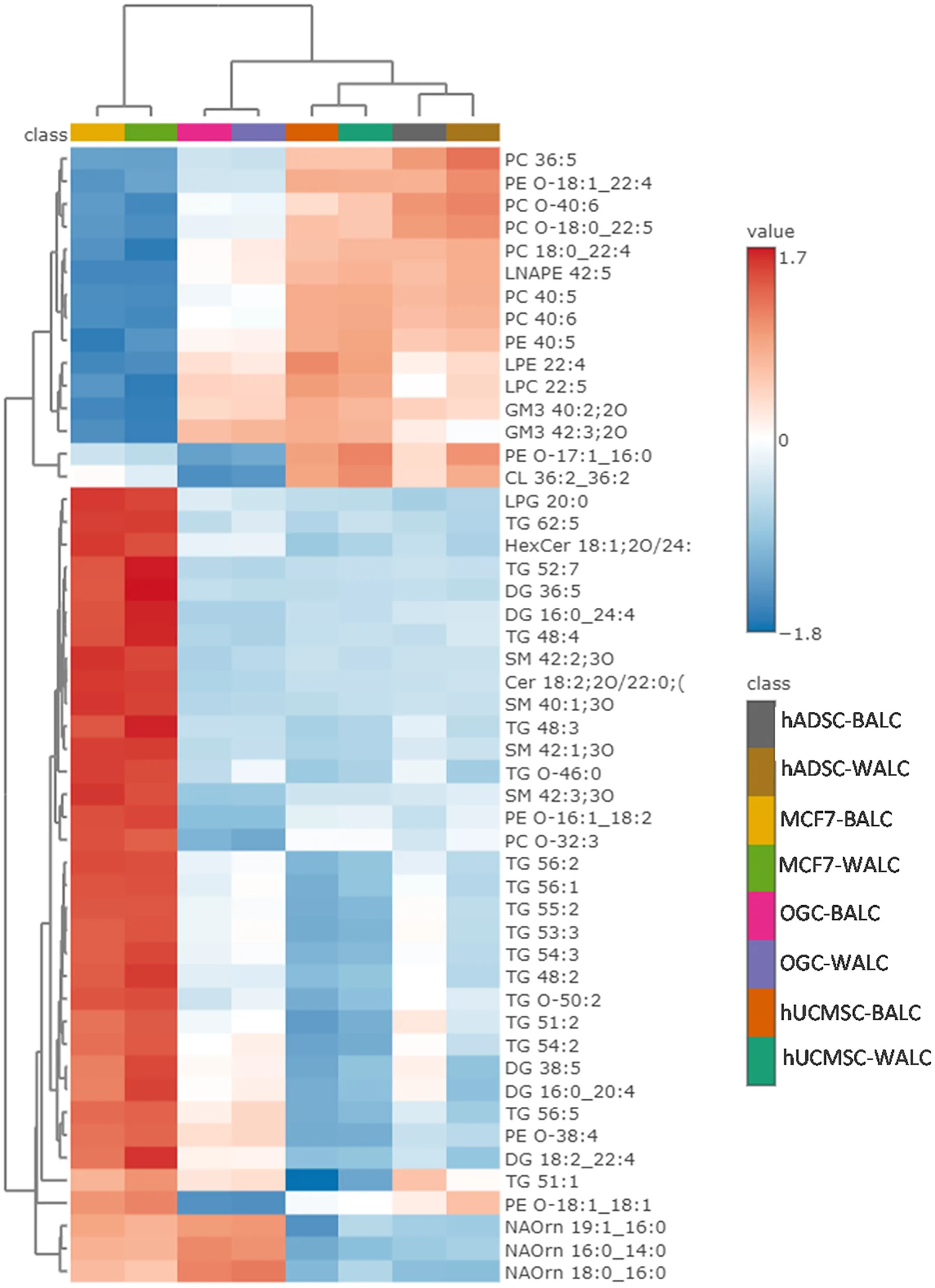
Hierarchical Clustering Heatmap of the top 50 lipid species for all treated cells. Lipids were extracted from exosome-treated cells and analyzed using LC–MS. The heatmap depicts the difference in levels of lipid species between different cells. Levels of normalized peak zones are displayed on the color scale. The color scale designates several standard deviations from the overall average of the lipid species. The zones in red reflect a higher amount of the lipid species, while the zones in blue reveal a lower amount. Standardization: Auto scale feature; Clustering method: Average; Fonts: 13; Annotation bar: 0.016; Show only group averages. The figure was generated using MetaboAnalyst 6.0.

Hierarchical clustering heatmap analysis was undertaken using Ward’s method with Euclidean distance (Fig 12). Regardless of treatment, similar cell types exhibited clustered lipidomic profiles. Nevertheless, hADSCs and hUCMSCs presented the highest degree of clustering, both with each other and with OGCs. MCF-7 breast cancer cells displayed the most distinct lipid profile compared to the other cell lines. Among the lipid species, phosphatidylcholine was found at the highest levels in hADSCs and hUCMSCs, while TGs were most abundant in MCF-7 breast cancer cells. These lipid species clustered separately, reflecting potential close metabolic links. Phosphatidylcholines and phosphatidylethanolamines were more abundant in hUCMSCs and less in MCF-7 breast cancer cells. Conversely, NAOrn lipids were enriched in OGCs but diminished in hUCMSCs and hADSCs. Ether-esterified lipids, however, exhibited a scattered distribution (Fig 12).

**Fig 13:**
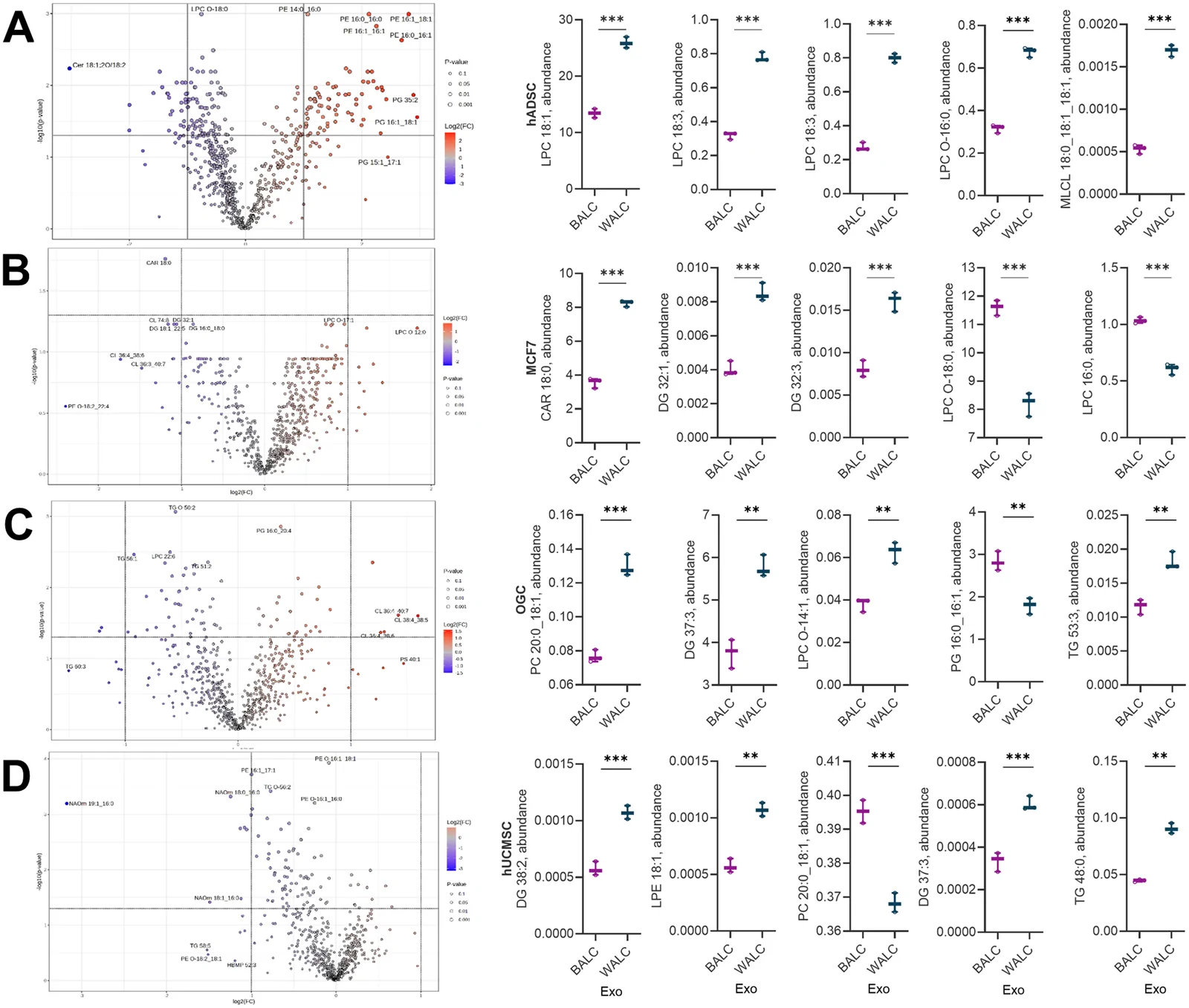
Cell type-specific lipidomic alterations following treatment with adipocyte-like cell-derived exosomes. Each row corresponds to one recipient cell type: human adipose-derived stem cells (hADSCs; **A**), MCF-7 breast cancer cells (**B**), ovarian granulosa cells (OGCs; **C**), and human umbilical cord mesenchymal stem cells (hUCMSCs; **D**). For each cell type, the volcano plot summarizes differential lipid abundance between brown adipocyte-like cell-derived exosome-treated cells (BALC-Exo) and white adipocyte-like cell-derived exosome-treated cells (WALC-Exo). The x-axis represents log_2_ fold change, and the y-axis reflects –log_10_(p-value). Vertical dashed lines reveal |log_2_FC| ≥ 1, equivalent to a two-fold change, and the horizontal dashed line demonstrates p < 0.05. Positive log_2_FC values denote enrichment after BALC-Exo treatment, whereas negative values signal diminished abundance relative to WALC-Exo treatment. The five box plots display the abundance of the top five altered lipid species identified in the corresponding cell type.

The volcano plots for each cell line indicate cell-specific responses to the treatment (Fig 13). hADSCs exhibited a relatively high number of differentially expressed lipids, with more upregulated hits, suggesting a pronounced impact of the treatment on this cell type. In contrast, hUCMSCs revealed the least change in response to the various exosome treatments.

In hADSCs, exposure to BALC-derived exosomes increased several phospholipid species, particularly phosphatidylethanolamines (PE 16:0_18:1, PE 16:0_16:0) and phosphatidylglycerols (PG 35:2, PG 16:1_18:1, PG 15:1_17:1). In parallel, a marked downregulation of long-chain ceramides (Cer 18:1/22:0 and Cer 18:1/24:1) was observed, signaling a potential shift from sphingolipid- to glycerophospholipid-enriched membranes.

MCF-7 breast cancer cells exhibited a broad lipid enrichment following treatment with BALC-derived exosomes, including ceramides (Cer 18:0), multiple cardiolipins (CL 74:6, CL 74:5, CL 72:5, CL 74:8, CL 74:7), and diglycerides (DG 18:1_22:0, DG 18:0_22:0), suggesting augmented mitochondrial lipid remodeling and neutral lipid metabolism. In contrast, key membrane lipids such as phosphatidylcholines (PC O-18:2_22:4) and lysophosphatidylcholines (LPC 17:0, LPC 18:0) were downregulated, reflecting possible alterations in membrane fluidity or turnover.

In OGCs, treatment with BALC-derived exosomes predominantly suppressed triglyceride species (e.g., TG 50:2, TG 50:1) while elevating mitochondrial-associated cardiolipins (CL 38:4, CL 38:5) and phosphatidylglycerol (PG 38:6). This inverse regulation suggests a metabolic shift away from lipid storage toward mitochondrial membrane specialization, potentially associated with steroidogenic function.

hUCMSCs responded to BALC-derived exosomes with upregulation of ether-linked and plasmalogen phospholipids (e.g., NAOm 19:1_16:0, NAOm 19:1_18:0, PE-O 34:1_38:0, PE 16:1_18:1), highlighting changes in redox-sensitive lipid signaling pathways. Concurrently, several triglycerides (TG 51:1, TG 51:2) and PE-O 18:2_18:1 were significantly reduced, again suggesting a coordinated downregulation of storage lipids.

## Discussion

Adipose tissue as an active secretory organ generates extracellular vesicles contributing to intercellular communication. Because brown and white adipocytes are different in metabolic function, their secreted exosomes are expected to exert distinct biological effects [3]. This study provided the first integrated evaluation of crude CM and isolated exosomes from brown and white adipocyte-like cells on stemness, differentiation, and metabolic reprogramming across four human cell types. Lipidomic data can offer insights into how adipocyte-derived exosomes may influence intercellular metabolic communication across distinct biological contexts.

In ovarian cells, treatment with BALC-derived exosomes boosted the expression of key stemness transcription factors Sox2 and Oct4, suggesting a rejuvenated fertility phenotype. Brown adipose tissue-derived exosomes can transport bioactive molecules that modulate signaling pathways in ovarian cells [34,35]. The functional effects of BALC-derived exosomes were accompanied by augmented estradiol secretion, aligning with previous findings that exosomes derived from pluripotent stem cell–MSCs or umbilical cord MSCs can restore hormone production and reduce apoptosis in ovarian insufficiency models [36,37].

In the present study, lipidomics analysis revealed elevated levels of cardiolipins (CL 38:4, CL 38:5) and phosphatidylglycerol (PG 38:6) in BALC- exosome–treated OGCs. As these lipid classes are closely tied to mitochondrial membranes, these changes may indicate alterations in mitochondrial membrane composition rather than directly proving enhanced mitochondrial respiration [38]. The concomitant reduction in triglyceride (TG) species, including TG 50:2 and TG 50:1, further suggests a shift in lipid handling away from storage-associated lipids. Nevertheless, whether these lipidomic changes reflect altered oxidative phosphorylation, steroidogenic metabolism, or mitochondrial activity requires validation through functional metabolic assays.

A decline in the expression of stemness-associated genes or lose of stemness could disturb differentiation and stemness properties of the MSC population in obese models [39]. In hADSCs, BALC-derived exosomes lowered stemness markers while promoting osteogenic features, signifying a shift toward lineage commitment. Lipidomic profiling supported this phenotypic transition at the membrane and lipid-metabolism level. Elevated phosphatidylethanolamines, including PE 16:0_18:1 and PE 16:0_16:0, together with changes in cardiolipins, may reflect increased membrane remodeling linked to differentiation. The reduction of long-chain ceramides, such as Cer 18:1/22:0, may accord with reduced pro-apoptotic lipid signaling, though this interpretation remains correlative. Increased N-acyl lipids and lysophosphatidylethanolamines may also signal adaptive changes in lipid signaling and membrane turnover during the response to BALC-derived exosomes.

In MCF-7 breast cancer cells, both BALC- and WALC-derived exosomes lowered Sox2 and Oct4 expression, indicating suppression of stemness-associated features. Cancer stem-like cell populations contribute to lineage persistence, stress tolerance, and resistance to therapy [40]. CM from both adipocyte-like cell types by reducing Bcl-2 and increasing Bax and caspase-3 expressions, revealed effects consistent with activation of apoptosis-associated pathways. Lipidomic alterations indicated heightened cardiolipins and diglycerides, which may reflect changes in mitochondrial membrane composition or lipid remodeling during apoptosis-associated stress. In contrast, reduced carnitine-conjugated lipids may suggest altered fatty-acid-related metabolism, but these data alone do not establish diminished fatty acid oxidation capacity or mitochondrial dysfunction [41,42]. Reductions in phosphatidylcholine and lysophosphatidylcholine species further suggest changes in membrane composition that may influence cancer cell signaling and survival.

In hUCMSCs, exposure to BALC-derived exosomes was linked to increased ether-linked and plasmalogen phospholipids, suggesting changes in membrane composition, redox-sensitive lipid pathways, and lipid signaling. These changes were accompanied by lowered triglycerides and N-acyl ornithines, reflecting altered storage-lipid and stress-associated lipid profiles. Nevertheless, the interpretation that these changes represent reduced metabolic stress, energy-efficient programming, or preservation of regenerative capacity remains speculative without functional validation. Thus, the lipidomic signatures observed in hUCMSCs should be considered as indicators of altered lipid state rather than direct evidence of metabolic reprogramming [43].

Taken together, BALC- and WALC-derived exosomes induced cell-type-specific phenotypic and lipidomic responses. OGCs revealed heightened estradiol secretion together with changes in mitochondrial membrane-associated lipids; hADSCs indicated diminished stemness markers and enhanced osteogenic features; MCF-7 cells demonstrated reduced stemness markers and increased apoptosis-associated signaling; and hUCMSCs exhibited distinct changes in ether-linked, plasmalogen, and storage lipids. These findings suggest that adipocyte-derived exosomes may modulate stemness, differentiation, apoptosis, and lipid remodeling in a cell-type-specific manner. However, the lipidomic findings should be interpreted as hypothesis-generating. Functional assays assessing mitochondrial respiration, ATP production, reactive oxygen species, mitochondrial membrane potential, and fatty acid oxidation would be required to determine whether the observed lipid changes correspond to altered mitochondrial function or metabolic activity.

The primary aim of this study was to characterize cell-type-specific responses to adipocyte-derived exosome-enriched preparations rather than to dissect the underlying molecular mechanisms. The heterogeneous composition of CM and exosomes preparations restricts the attribution of the observed effects to specific molecular drivers.

The present work identified different functional effects of BALC- and WALC-derived conditioned media and exosomes linking to differential lipid signatures. Nevertheless, the limitation of our study was that specific molecular cargos responsible for mediating observed effects remained uninvestigated. We suggest that future studies capturing exosomal miRNA sequencing, proteomic profiling, and targeted functional validation should be performed. Such upcoming work can help identify the regulatory components directing adipocyte-derived paracrine signaling and establish fundamental mechanisms underlying the mentioned cellular responses.

## Conclusion

In conclusion, exosomes from BALCs and WALCs exert distinct, cell-specific effects compared with soluble factors, affecting lipid metabolism, differentiation, and apoptosis in vitro. These findings highlight a potential modulatory role of adipocyte-derived exosomes in intercellular communication. However, the conclusions are limited by the in vitro setting and the number of models examined, warranting further validation across in vivo contexts.

## Supporting information

S1 Table Primer sequences and RefSeq accession numbers for qRT-PCR analysis of target. (DOCX)

S2 Table Lipid classes. The different classes of lipids were summarized in Table.
(DOCX)

S1 Fig Standard curve of the BCA assay. The X axis concentration and Y axis: absorbance.
(TIFF)

S2 Fig Microscopic image of isolated ovarian granulosa cells in culture. A representative brightfield image showing isolated ovarian granulosa cells at passage 2, displaying their typical morphology and growth in cell culture.
(TIF)

S1 File Graphical abstract: This study explores how the unique collection of factors secreted by white versus brown adipocyte-like cells differentially influences target cell phenotypes and alters their lipid profiles. (TIF)
