# Supplementary material for: Brown and white adipocyte like cells derived exosomes differentially regulate stemness, hormone secretion, apoptosis, and lipid metabolism in model cells
Source: PLoS One. 2026 Sep 1;21(9):e0355955. doi: 10.1371/journal.pone.0355955 (PMC13533381; doi:10.1371/journal.pone.0355955)
Supplement: Supplementary file 1 [file pone.0355955.s001.docx]

**Supplementary Table 1**. Primer sequences and RefSeq accession numbers for qRT-PCR analysis of target genes.

| Gene | Forward (5′→ 3′) | Reverse (3′→ 5′) | Annealing temperature (℃) | NCBI Gene Accession |
| --- | --- | --- | --- | --- |
| UCP1 | GTGTGCCCAACTGTGCAATG | CCAGGATCCAAGTCGCAAGA | 58 | NM_021833.5 |
| PLIN1 | ACCCCCCTGAAAAGATTGCTT | GATGGGAACGCTGATGCTGTT | 58 | NM_002666.3 |
| GAPDH | TTGCCCTCAACGACCACTTT | TGGTCCAGGGGTCTTACTCC | 60 | NM_002046.7 |
| Bcl2 | ACTGGAGAGTGCTGAAGATTG | GTCTACTTCCTCTGTGATGTTGT | 60 | NM_000633.3 |
| Bax | GGTCTTTTTCCGAGTGGCAG | CCTTGAGCACCAGTTTGCTG | 60 | NM_138761.4 |
| Caspase3 | AGCGAATCAATGGACTCTGG | CCGAGATGTCATTCCAGTGC | 60 | NM_004346.4 |
| RUNX2 | TCATGGCGGGTAACGATGA | GGGAGGATTTGTGAAGACGG | 60 | NM_001278478.2 |
| Osteocalcin | CCT CAC ACT CCT CGC CCT ATT | GGT CAG CCA ACT CGT CACA | 60 | NM_199173.6 |
| Osteopontin | GCC GAG GTG ATA GTG TGG TT | AAC GGG GAT GGC CTT GTA TG | 60 | NM_000582.3 |
