# Supplementary material for: Brown and white adipocyte like cells derived exosomes differentially regulate stemness, hormone secretion, apoptosis, and lipid metabolism in model cells
Source: PLoS One. 2026 Sep 1;21(9):e0355955. doi: 10.1371/journal.pone.0355955 (PMC13533381; doi:10.1371/journal.pone.0355955)
Supplement: Supplementary file 2 [file pone.0355955.s002.docx]

**Supplementary Table 2.** Lipid classes.

| **Abbreviation** | **Lipid Class** |
| --- | --- |
| CAR | Acylcarnitines |
| CE | Cholesteryl ester |
| Cer | Ceramides |
| CL | Cardiolipin |
| CoQ | Coenzym Q |
| DG | Diacylglycerides |
| FA | (Free) Fatty acid |
| GM3 | Ganglioside GM3 |
| HBMP | Hemibismonoacylglycerophosphate |
| HexCer | Hexosylceramide/Cerebroside |
| Hex2Cer | Dihexosylceramide |
| Hex3Cer | Trihexosylceramide |
| LNAPE | N-acyl lysophosphatidylethanolamine |
| LPA | Lysophophatidic acid |
| LPC | Lysophosphatidylcholine |
| LPC O | Ether-linked lysophosphatidylcholine/plasmalogen LPC |
| LPE | Lysophosphatidylethanolamine |
| LPE O | Ether-linked lysophosphatidylethanolamine/plasmalogen LPE |
| LPG | Lysophosphatidylglycerol |
| LPI | Lysophosphatidylinositol |
| LPS | Lysophosphatidylserine |
| MLCL | Lysocardiolipin |
| NAOrn | N-acyl ornithine |
| PC | Phosphatidylcholine |
| PC O | Ether-linked phosphatidylcholine/Plasmalogen PC |
| PE | Phosphatidylethanolamine |
| PE O | Ether-linked phosphatidylethanolamine/Plasmalogen PE |
| PE-Cer | Ceramide phosphatidylethanolamine |
| PG | Phosphatidylglycerol |
| PG O | Ether-linked phosphatidylglycerol/Plasmalogen PG |
| PI | Phosphatidylinositol |
| PS | Phosphatidylserine |
| SM | Sphingomyelin |
| ST | Cholesterol sulfate |
| TG | Triacylglyceride |
| TG O | Ether-linked triacylglyceride |
