# Supplementary material for: Brown and white adipocyte like cells derived exosomes differentially regulate stemness, hormone secretion, apoptosis, and lipid metabolism in model cells
Source: PLoS One. 2026 Sep 1;21(9):e0355955. doi: 10.1371/journal.pone.0355955 (PMC13533381; doi:10.1371/journal.pone.0355955)
Supplement: Supplementary file 3 [file pone.0355955.s006.docx]

4/19/2026

Dear Editor,

Thank you for your careful assessment of our manuscript and for highlighting the need to clarify the recruitment period.

In response to your comment, we have revised the Methods section to explicitly state the start and end dates of the recruitment and human sample collection period in the required DD/MM/YYYY format. Specifically, we have added the following sentence to the Ethics statement in Methods section (page 5):

“The recruitment and collection of human samples for this study were conducted from 28/06/2021 to 03/07/2023.”

We hope that this revision satisfactorily addresses your concern. We appreciate your guidance in improving the clarity and completeness of our manuscript.

Sincerely,

Effat Alizadeh (PhD), Associate Professor; corresponding author

Full postal address: Department of Medical Biotechnology, Faculty of Advanced Medical Sciences, Tabriz University of Medical Sciences, Tabriz, Iran.

Tel: +98 (41) 33377644- Cellphone: +989144059856,

E-mail address: Alizadehe@tbzmed.ac.ir – e.alizadeh.2010@gmail.com
