# Supplementary material for: Brown and white adipocyte like cells derived exosomes differentially regulate stemness, hormone secretion, apoptosis, and lipid metabolism in model cells
Source: PLoS One. 2026 Sep 1;21(9):e0355955. doi: 10.1371/journal.pone.0355955 (PMC13533381; doi:10.1371/journal.pone.0355955)
Supplement: Supplementary file 4 [file pone.0355955.s007.docx]

07/07/2026

Manuscript Number: PONE-D-26-16792

Submission of revise,

**Dear Professor Editor/s,**

Thank you very much for giving us the opportunity to submit a revised version of our manuscript entitled “**Brown and white adipocyte like cells derived exosomes differentially regulate stemness, hormone secretion, apoptosis, and lipid metabolism in model cells”** for publication in the esteemed Journal **PLOS One**. We appreciate the time and efforts that editor and the reviewers dedicated to provide feedback on our manuscript and are grateful for the insightful comment on and valuable improvements to our paper. We incorporated the suggestions made by the reviewer. Please see below, for a point-by-point response to the editor/s and reviewer/s comment. Also, we uploaded a revised version changes highlighted/track changed as well as a clean revised manuscript. Thank you in advance. We look forward to hearing from you.

Sincerely,

Dr. Effat Alizadeh,

Associate Professor, Department of Medical Biotechnology,

Faculty of Advanced Medical Sciences, Tabriz University of Medical Sciences, Tabriz, Iran

Phone: +984133341933

Mobile: +989144059856

[Alizadehe@tbzmed.ac.ir](mailto:Alizadehe@tbzmed.ac.ir) or [e.alizadeh.2010@gmail.com](mailto:e.alizadeh.2010@gmail.com)

ORCID codes of Authors:

Zeinab Ghesmati (ORCiD: 0000-0002-8416-2298)

Mohsen Rashid (ORCiD: 0000-0003-2042-1083)

Shabnam Fayezi (ORCiD: 0000-0001-6954-5138)

Effat Alizadeh (ORCiD: 0000-0002-7481-0896)

Masoud Darabi (ORCiD: 0000-0001-6380-272X)

Response to comments:

**1.**The manuscript is of interest pending some points to be revised, including a more insight on the specific exosomal components driving these changes and vesicle characterization by assessing an endoplasmic reticulum marker (such as Calnexin or Calreticulin) to rule out cellular contamination. This will make more robust the findings

Response: We thank the Academic Editor for this valuable suggestion. The present study was designed as an *in vitro* functional and lipidomic analysis of adipocyte-derived exosome-enriched preparations from brown and white adipocyte-like cells, rather than a comprehensive molecular cargo discovery study of exosomes. We have now acknowledged this limitation in the Discussion (Page number: 39) and added that future proteomic and transcriptomic analyses, including miRNA profiling, will be required to identify candidate molecular mediators and validate their functional contribution.

Calnexin, as a negative marker, has now been included in the additional characterization experiments for both groups (BALC‑Exo and WALC‑Exo) by Western blotting. The revised Methods (Page number: 12), Results, and Figure 5G (Page numbers: 21-22) now indicate that the isolated exosome preparations were positive for CD9, CD63, and TSG101 and negative for calnexin.

Journal Requirements:

**1.** Please ensure that your manuscript meets PLOS ONE's style requirements, including those for file naming. The PLOS ONE style templates can be found at

https://journals.plos.org/plosone/s/file?id=wjVg/PLOSOne_formatting_sample_main_body.pdf and https://journals.plos.org/plosone/s/file?id=ba62/PLOSOne_formatting_sample_title_authors_affiliations.pdf

Response: Thank you for this comment. We have carefully reviewed the manuscript and supporting files to ensure compliance with PLOS ONE style requirements, including file naming conventions and formatting guidelines. The manuscript and associated files have been revised accordingly and prepared in accordance with the PLOS ONE style templates.

**2.** Please provide additional details regarding participant consent. In the ethics statement in the Methods and online submission information, please ensure that you have specified (1) whether consent was informed and (2) what type you obtained (for instance, written or verbal, and if verbal, how it was documented and witnessed). If your study included minors, state whether you obtained consent from parents or guardians. If the need for consent was waived by the ethics committee, please include this information. If you are reporting a retrospective study of medical records or archived samples, please ensure that you have discussed whether all data were fully anonymized before you accessed them and/or whether the IRB or ethics committee waived the requirement for informed consent. If patients provided informed written consent to have data from their medical records used in research, please include this information.

Response:

Thank you very much for this important comment. We have revised the Ethics Statement in the Methods section to provide additional details regarding participant consent. Specifically, we clarified that informed consent was obtained from all participants prior to sample collection and study participation. We also specified the type of consent obtained, indicating that written informed consent was collected in accordance with the requirements of the approving ethics committee. Participation was voluntary, and all procedures complied with institutional guidelines and the principles of the Declaration of Helsinki. Where applicable, we additionally clarified that no minors were included in this study; therefore, parental or guardian consent was not required. No waiver of informed consent was requested or granted by the ethics committee. The revised ethics statement has been updated accordingly in the manuscript.

**3.** Please note that PLOS One has specific guidelines on code sharing for submissions in which author-generated code underpins the findings in the manuscript. In these cases, we expect all author-generated code to be made available without restrictions upon publication of the work. Please review our guidelines at https://journals.plos.org/plosone/s/materials-and-software-sharing#loc-sharing-code and ensure that your code is shared in a way that follows best practice and facilitates reproducibility and reuse.

Response: Thank you for bringing this to our attention. We reviewed the PLOS ONE guidelines regarding code sharing and confirm that no author-generated code was developed or used to generate, process, analyze, or interpret the findings presented in this manuscript. All analyses were performed using standard commercially available and/or publicly accessible software with their built-in functions and settings. Accordingly, there is no custom code associated with this study that requires public deposition.

**4.** Please update your submission to use the PLOS LaTeX template. The template and more information on our requirements for LaTeX submissions can be found at <http://journals.plos.org/plosone/s/latex>.

Response: Thank you for your comment and for providing the PLOS formatting guidance. We understand the recommendation regarding use of the PLOS LaTeX template. However, we do not have an existing LaTeX version of the manuscript available. Therefore, we have retained the manuscript in its current format and ensured that it complies with all applicable journal formatting and submission requirements.

**5.** Thank you for stating the following in the Acknowledgments Section of your manuscript:

“This work was part of the first author's Ph.D. dissertation, completed under the supervision of Effat Alizadeh and Masoud Darabi. This study was supported by the Tabriz University of Medical Sciences, Tabriz, Iran (Grant No. 66664) and the University Medical Center Schleswig-Holstein, Lübeck, Germany.” We note that you have provided funding information that is not currently declared in your Funding Statement. However, funding information should not appear in the Acknowledgments section or other areas of your manuscript. We will only publish funding information present in the Funding Statement section of the online submission form.  Please remove any funding-related text from the manuscript and let us know how you would like to update your Funding Statement. Currently, your Funding Statement reads as follows: “This work was part of the first author's Ph.D. dissertation, completed under the supervision of Effat Alizadeh and Masoud Darabi. This study was supported by the Tabriz University of Medical Sciences, Tabriz, Iran (Grant No. 66664) and the University Medical Center Schleswig-Holstein, Lübeck, Germany.”Please include your amended statements within your cover letter; we will change the online submission form on your behalf.

Response:

We greatly appreciate your clarification regarding the presentation of funding information. We acknowledge that funding-related information was inadvertently included in the manuscript text. In accordance with PLOS ONE requirements, we have removed all funding-related statements from the manuscript and retained only the non-funding acknowledgment text.We request that the Funding Statement in the online submission system be amended to include only the funding information as follows:

“This study was supported by the Tabriz University of Medical Sciences, Tabriz, Iran (Grant No. 66664) and the University Medical Center Schleswig-Holstein, Lübeck, Germany.”

The following statement has been retained in the manuscript Acknowledgments section without amendment, as it is not a funding declaration:

“This work was part of the first author's Ph.D. dissertation, completed under the supervision of Effat Alizadeh and Masoud Darabi.”

Please update the online submission form accordingly.

**6.** Thank you for stating the following financial disclosure:

“This work was part of the first author's Ph.D. dissertation, completed under the supervision of Effat Alizadeh and Masoud Darabi. This study was supported by the Tabriz University of Medical Sciences, Tabriz, Iran (Grant No. 66664) and the University Medical Center Schleswig-Holstein, Lübeck, Germany.” Please state what role the funders took in the study.  If the funders had no role, please state: "The funders had no role in study design, data collection and analysis, decision to publish, or preparation of the manuscript." If this statement is not correct you must amend it as needed. Please include this amended Role of Funder statement in your cover letter; we will change the online submission form on your behalf.

Response: Many thanks to request for clarification regarding the role of the funders.

We confirm that the funding organizations provided financial support for conducting the study but had no involvement in the scientific or publication-related aspects of the work. Accordingly, we request that the Role of Funder statement in the online submission system be updated as follows: “The funders had no role in study design, data collection and analysis, decision to publish, or preparation of the manuscript.” Please update the online submission form accordingly.

**7.** Thank you for stating the following financial disclosure:

“This work was part of the first author's Ph.D. dissertation, completed under the supervision of Effat Alizadeh and Masoud Darabi. This study was supported by the Tabriz University of Medical Sciences, Tabriz, Iran (Grant No. 66664) and the University Medical Center Schleswig-Holstein, Lübeck, Germany.” We note that one or more of the authors is affiliated with the funding organization, indicating the funder may have had some role in the design, data collection, analysis or preparation of your manuscript for publication; in other words, the funder played an indirect role through the participation of the co-authors. If the funding organization did not play a role in the study design, data collection and analysis, decision to publish, or preparation of the manuscript and only provided financial support in the form of authors' salaries and/or research materials, please do the following:

1. Review your statements relating to the author contributions, and ensure you have specifically and accurately indicated the role(s) that these authors had in your study. These amendments should be made in the online form.

2. Confirm in your cover letter that you agree with the following statement, and we will change the online submission form on your behalf: 
“The funder provided support in the form of salaries for authors [insert relevant initials], but did not have any additional role in the study design, data collection and analysis, decision to publish, or preparation of the manuscript. The specific roles of these authors are articulated in the ‘author contributions’ section.

Response: Thank you for your clarification regarding disclosure of the funder’s role and author affiliations. We have reviewed the Author Contributions statements and ensured that the contributions of all authors are specifically and accurately reflected in the online submission information. We confirm that the funding organizations provided support solely in the form of research funding. Furthermore, the funding organizations had no additional role in the study design, data collection and analysis, decision to publish, or preparation of the manuscript. Any contributions made by authors affiliated with the funding organizations were performed independently in their capacity as researchers and are explicitly described in the Author Contributions section. Accordingly, we request that the online submission form be updated to reflect this clarification.

**8.** PLOS ONE now requires that authors provide the original uncropped and unadjusted images underlying all blot or gel results reported in a submission’s figures or Supporting Information files. This policy and the journal’s other requirements for blot/gel reporting and figure preparation are described in detail at https://journals.plos.org/plosone/s/figures#loc-blot-and-gel-reporting-requirements and https://journals.plos.org/plosone/s/figures#loc-preparing-figures-from-image-files. When you submit your revised manuscript, please ensure that your figures adhere fully to these guidelines and provide the original underlying images for all blot or gel data reported in your submission. See the following link for instructions on providing the original image data: https://journals.plos.org/plosone/s/figures#loc-original-images-for-blots-and-gels.    In your cover letter, please note whether your blot/gel image data are in Supporting Information or posted at a public data repository, provide the repository URL if relevant, and provide specific details as to which

Response:

Many thanks, we have reviewed the journal guidelines and ensured that all figures included in the revised submission comply with the requirements for blot/gel reporting and figure preparation. The original uncropped and unadjusted images underlying all blot and gel results reported in the manuscript have been provided as Supporting Information files to facilitate transparency, reproducibility, and assessment of image integrity. No modifications beyond those described in the manuscript were applied to the original source images.

**9.** Your ethics statement should only appear in the Methods section of your manuscript. If your ethics statement is written in any section besides the Methods, please move it to the Methods section and delete it from any other section. Please ensure that your ethics statement is included in your manuscript, as the ethics statement entered into the online submission form will not be published alongside your manuscript.

Response: We have reviewed the manuscript and ensured that the ethics statement appears only within the Methods section in accordance with PLOS ONE requirements. Any duplicate ethics-related text appearing elsewhere in the manuscript has been removed.

**10.** We notice that your supplementary figures are included in the manuscript file. Please remove them and upload them with the file type 'Supporting Information'. Please ensure that each Supporting Information file has a legend listed in the manuscript after the references list.

Response: Thank you for bringing this to our attention. We have removed the supplementary figures from the main manuscript file and uploaded them separately under the appropriate file type, “Supporting Information,” in accordance with the journal requirements. Additionally, legends for each Supporting Information file have been added to the manuscript and placed after the references section as requested.

11. If the reviewer comments include a recommendation to cite specific previously published works, please review and evaluate these publications to determine whether they are relevant and should be cited. There is no requirement to cite these works unless the editor has indicated otherwise. 

Response: We greatly appreciate this clarification regarding reviewer-suggested citations.

We confirm that the reviewer comments did not include recommendations to cite any specific previously published works. Therefore, no additional evaluation or citation additions were required in response to this request. The manuscript references remain unchanged except where revisions were otherwise necessary.

**Reviewer #1:**

1-The study explores a highly relevant topic in metabolic and regenerative medicine: the paracrine signaling of brown and white adipocytes via exosomes. The use of four different human cell models (OGCs, MCF-7, hADSCs, hUCMSCs) provides a comprehensive overview of how these secretomes influence diverse biological processes, from cancer apoptosis to fertility and stemness. The integration of lipidomic profiling adds significant depth to the phenotypic observations.The characterization of isolated hADSCs and their subsequent differentiation into BALCs and WALCs is well-documented using flow cytometry, qPCR, and Western Blotting. The study doesn't limit itself to phenotypic changes but seeks a metabolic explanation through mass spectrometry-based lipidomics. The differential effects (e.g., BALCs-Exo increasing estradiol in OGCs while CM reduces it) are striking and suggest complex regulatory mechanisms. While the paper establishes what happens, the how remains largely correlative. Identifying specific exosomal cargo (miRNAs or proteins) responsible for the observed effects would significantly strengthen the impact.

Response: We appreciate the respected reviewer for the recognition of the novelty and translational relevance of our study, particularly the integration of adipocyte like cells-derived secretome biology with functional and lipidomic analyses across multiple human cell models. We agree that our results demonstrate strong biological effects of conditioned media and exosomes derived from BALCs and WALCs, the molecular mediators responsible for them remain totally unknown. The aim of current study was a comprehensive functional and phenotypic investigation to determine whether adipocyte-derived extracellular signaling exerts distinct regulatory effects across reproductive, cancer, and stem cell contexts and whether these effects are linked to the variances in lipid composition. Our results indicate that the observed responses cannot be explained solely by global secretome exposure, as notable differences were detected between conditioned media and purified exosomes (for example, BALCs-Exo increased estradiol secretion in OGCs whereas corresponding conditioned media reduced this response). These findings support the concept that selective cargo packaging within extracellular vesicles may generate distinct downstream signaling outcomes. We fully acknowledge that characterization of exosomal cargo—including miRNAs, proteins, and additional bioactive molecules—would substantially strengthen mechanistic interpretation and represents an important next step. Candidate mechanisms may involve differential enrichment of adipocyte-associated regulatory molecules that influence steroidogenesis, apoptosis, metabolic reprogramming, and stemness pathways. However, comprehensive cargo profiling and functional validation (e.g., small RNA sequencing, proteomics, cargo depletion, gain/loss-of-function approaches, or pathway inhibition studies) were beyond the scope of the present work. To address this point, we have added the following statement to the Discussion section:

“The present work identified different functional effects of BALC- and WALC-derived conditioned media and exosomes linking to differential lipid signatures. However, the limitation of our study is that specific molecular cargos responsible for mediating observed effects remains uninvestigated. We suggest that future studies including exosomal miRNA sequencing, proteomic profiling, and targeted functional validation should be performed. Such upcoming work can help to identify the regulatory components directing adipocyte-derived paracrine signaling and establish fundamental mechanisms underlying mentioned cellular responses.” We believe this clarification strengthens the interpretation of our findings and better positions the present work as a mechanistic foundation for future cargo-resolved investigations.

2-Moreover, for vesicle characterization, endoplasmic markers such as calreticulin and/or calnexin should be added to exclude contamination of vesicles derived from the endoplasmic reticulum.

**Response:** We greatly appreciate the reviewer ‘s comment. Calnexin, as a negative marker, has now been included in the additional characterization experiments for both groups (BALC‑Exo and WALC‑Exo) by Western blotting. The revised Methods (Page number: 12) Results, and Figure 5G (Page numbers: 21-22now indicate that the isolated exosome preparations were positive for CD9, CD63, and TSG101 and negative for calnexin.

3-In figure 7A, change “Annexin 3” with “Annexin V”.

**Response:** We thank the reviewer for carefully noting this issue. The label has been corrected from ‘Annexin 3’ to ‘Annexin V’ in the revised version of the manuscript (Figure 8; Page number: 26).

4-Then, the discussion should be more concise, prioritizing the interpretation of key findings and expanding the speculation regarding the potential molecular/proteins content of the vesicles.

**Response**: Many thanks to the reviewer for this valuable suggestion. In the revised manuscript, the Discussion section has been condensed (from 964 words in submitted version reached 830 words in revised version) and revised to focus more directly on the interpretation of the principal cell-type–specific findings while avoiding overinterpretation of the descriptive lipidomic data. We have also added a focused paragraph discussing potential exosome-associated mediators, including miRNAs, proteins, and lipid cargo, while clearly presenting these as hypotheses that require future validation. We also acknowledge that the precise identification of the active vesicular components remains a limitation of the current study and warrants further investigation in future studies (Page number: 39). We reduced word count of discussion from 905 to 874

Reviewer #2:

This manuscript investigates the effects of conditioned media (CM) and exosomes derived from brown adipocyte-like cells (BALCs) and white adipocyte-like cells (WALCs) on different human cell models, including ovarian granulosa cells (OGCs), MCF-7 breast cancer cells, hADSCs, and hUCMSCs. The topic is relevant and potentially interesting because adipocyte-derived extracellular vesicles are increasingly recognized as mediators of intercellular communication. The inclusion of lipidomics also increases the translational value of the study. However, although the manuscript contains potentially valuable observations, the current version remains descriptive and preliminary.

**Response:** We sincerely thank the reviewer for the thoughtful and constructive evaluation of our work and for recognizing both the relevance of adipocyte-derived extracellular vesicles as mediators of intercellular communication and the translational value of incorporating lipidomic analyses. We agree that the initial version of the manuscript was primarily observational and required stronger interpretation and contextualization. In response to this valuable comment, we revised the manuscript to improve scientific depth and clarify the biological significance of our findings.

1-In particular, the exosome characterization, mechanistic validation, and biological rigor require substantial improvement before the manuscript can be considered suitable for publication. The authors identify the isolated particles as “exosomes”, but the characterization is incomplete according to current extracellular vesicle (EV) guidelines (MISEV recommendations). Only morphology (TEM/SEM), size, zeta potential, and positive markers (CD9, CD63, TSG101) are shown. The evidence is not sufficient to exclude contamination from proteins, apoptotic bodies, lipoproteins, or other EV populations, especially because a precipitation kit was used for isolation. The authors should add:

- Nanoparticle tracking analysis (NTA) to determine particle concentration and size distribution.
- Negative EV markers (e.g., calnexin, GM130, or albumin) to demonstrate purity.
- Quantification of exosome yield and normalization strategy (particle number or protein amount).

**Response:** We thank the reviewer for these valuable and constructive suggestions. We agree that nanoparticle tracking analysis (NTA) is a useful method for determining particle size distribution and concentration in exosome preparations, as recommended by the MISEV guidelines. In the present study, exosomes were characterized using multiple complementary approaches, including electron microscopy to evaluate vesicle morphology, measurement of particle size and zeta potential, and detection of established exosomal markers (CD9, CD63, and TSG101). In addition, a negative marker was included to further support the purity of the vesicle preparations. While NTA-based quantification would provide additional information regarding particle concentration and size distribution, access to the specialized instrumentation required for this analysis was not available during the course of this study and the present revision. Future studies incorporating NTA analysis, in accordance with MISEV recommendations, will provide a more comprehensive characterization of the isolated exosomes.

Calnexin, as a negative marker, has now been included in the additional characterization experiments for both groups (BALC‑Exo and WALC‑Exo) by Western blotting. The revised Methods ((Page number: 12), Results, and Figure 5G ((Page numbers: 21-22) now indicate that the isolated exosome preparations were positive for CD9, CD63, and TSG101 and negative for calnexin. In the present study, the yield of isolated exosomes was estimated by measuring total protein concentration using a BCA protein assay. Standard curve of the BCA assay is shown in **Supplementary Fig 1** (Page number: 21)**.** For subsequent functional experiments, exosome preparations were normalized based on exosomal protein content to ensure equal dosing across experimental groups. All treatments were performed using an equal exosome protein concentration of 20 µg/mL (Page number: 12). The relevant sections in the Methods (Page number:11) and Results (Page number: 21) have been revised accordingly for clarity.

2.The manuscript assumes that target cells respond directly to adipocyte-derived exosomes, but there is no evidence that exosomes are internalized by recipient cells. The authors must perform exosome uptake assay using fluorescent labeling (e.g., PKH67, DiI, or membrane dye) and microscopy or flow cytometry.

**Response:** We thank the reviewer for this important comment. To address this concern, we performed an exosome uptake assay to confirm the internalization of exosomes derived from WALCs and BALCs by recipient cells. Exosome uptake was evaluated in the hADSCs group as a representative recipient cell population. The experimental procedure has been added to the revised *Methods* section (Page number:13), and the corresponding results and representative images are now included in the *Results* section (Page number: 23) and presented in the revised Figure 6 (Page number: 23)

3.The lipidomics section is interesting but highly speculative. Many conclusions regarding mitochondrial remodeling, oxidative phosphorylation, membrane specialization, or metabolic programming are not experimentally validated. To support these claims, authors should include functional metabolic assays, such as:

- Mitochondrial respiration analysis (Seahorse assay),
- ATP production assay,
- ROS measurements,
- Mitochondrial membrane potential assay.

**Response:** Many more thanks to the reviewer for this insightful comment. We agree that lipidomic changes alone cannot prove altered mitochondrial respiration, oxidative phosphorylation, ROS production or membrane-potential changes. We have therefore revised the Results (Figure 13; Page number: 35) and Discussion (Page number: 38) to present these lipidomic findings as hypothesis-generating rather than mechanistically proven. Statements referring to mitochondrial remodeling, oxidative phosphorylation and metabolic programming have been softened, and we now state that functional metabolic assays, including mitochondrial respiration, ATP production, ROS and mitochondrial membrane-potential measurements, will be required in future studies to validate these interpretations.

4.The osteogenic differentiation conclusion relies mainly on ALP activity. However, ALP is an early marker and does not fully demonstrate osteogenic commitment. The authors should add Alizarin Red quantification and evaluation of osteogenic genes (RUNX2, Osteocalcin, Osteopontin).

**Response**: We thank the reviewer for these valuable comments. We have now added Alizarin Red quantification to assess mineralization and evaluated osteogenic marker genes, including RUNX2, osteocalcin and osteopontin, by qRT-PCR. These results have been incorporated into the revised Methods (Page number: 15), Results (Page number: 29) and Figure 9B, 9C (Page number:27- 29) sections.

5.English should be revised by a native speaker.

**Response**: We greatly appreciate respected reviewer. The manuscript has been carefully revised for grammar, clarity, and scientific style, and the necessary corrections have been made throughout the revised version. **An English language editing certificate** has also been uploaded as part of the revised submission for your reference.
