# Supplementary material for: Brown and white adipocyte like cells derived exosomes differentially regulate stemness, hormone secretion, apoptosis, and lipid metabolism in model cells
Source: PLoS One. 2026 Sep 1;21(9):e0355955. doi: 10.1371/journal.pone.0355955 (PMC13533381; doi:10.1371/journal.pone.0355955)
Supplement: Peer review history

# Author comment: Author response to Decision Letter 0

**DOI:** 10.1371/journal.pone.0355955.r001

19 Apr 2026

Attachment Submitted filename: Response.docx

# Peer review: Decision Letter 0

**DOI:** 10.1371/journal.pone.0355955.r002

**Authors:** Gianpaolo Papaccio

11 May 2026

Dear Dr. Alizadeh,

Thank you for submitting your manuscript to PLOS ONE. After careful consideration, we feel that it has merit but does not fully meet PLOS ONE’s publication criteria as it currently stands. Therefore, we invite you to submit a revised version of the manuscript that addresses the points raised during the review process.

Please submit your revised manuscript by Jun 25 2026 11:59PM. If you will need more time than this to complete your revisions, please reply to this message or contact the journal office at plosone@plos.org. When you're ready to submit your revision, log on to https://www.editorialmanager.com/pone/ and select the 'Submissions Needing Revision' folder to locate your manuscript file.

- A letter that responds to each point raised by the academic editor and reviewer(s). You should upload this letter as a separate file labeled 'Response to Reviewers'.
- A marked-up copy of your manuscript that highlights changes made to the original version. You should upload this as a separate file labeled 'Revised Manuscript with Track Changes'.
- An unmarked version of your revised paper without tracked changes. You should upload this as a separate file labeled 'Manuscript'.

If you would like to make changes to your financial disclosure, please include your updated statement in your cover letter. Guidelines for resubmitting your figure files are available below the reviewer comments at the end of this letter.

If applicable, we recommend that you deposit your laboratory protocols in protocols.io to enhance the reproducibility of your results. Protocols.io assigns your protocol its own identifier (DOI) so that it can be cited independently in the future. For instructions see: https://journals.plos.org/plosone/s/submission-guidelines#loc-laboratory-protocols. Additionally, PLOS ONE offers an option for publishing peer-reviewed Lab Protocol articles, which describe protocols hosted on protocols.io. Read more information on sharing protocols at https://plos.org/protocols?utm_medium=editorial-email&utm_source=authorletters&utm_campaign=protocols.

As the corresponding author, your ORCID iD is verified in the submission system and will appear in the published article. PLOS supports the use of ORCID, and we encourage all coauthors to register for an ORCID iD and use it as well. Please encourage your coauthors to verify their ORCID iD within the submission system before final acceptance, as unverified ORCID iDs will not appear in the published article. *Only*  the individual author can complete the verification step; PLOS staff *cannot*  verify ORCID iDs on behalf of authors.

We look forward to receiving your revised manuscript.

Kind regards,

Gianpaolo Papaccio, M.D., Ph.D.

Academic Editor

PLOS One

Journal Requirements:

When submitting your revision, we need you to address these additional requirements.

1. Please ensure that your manuscript meets PLOS ONE's style requirements, including those for file naming. The PLOS ONE style templates can be found at

https://journals.plos.org/plosone/s/file?id=wjVg/PLOSOne_formatting_sample_main_body.pdf and

https://journals.plos.org/plosone/s/file?id=ba62/PLOSOne_formatting_sample_title_authors_affiliations.pdf

2. Please provide additional details regarding participant consent. In the ethics statement in the Methods and online submission information, please ensure that you have specified (1) whether consent was informed and (2) what type you obtained (for instance, written or verbal, and if verbal, how it was documented and witnessed). If your study included minors, state whether you obtained consent from parents or guardians. If the need for consent was waived by the ethics committee, please include this information. If you are reporting a retrospective study of medical records or archived samples, please ensure that you have discussed whether all data were fully anonymized before you accessed them and/or whether the IRB or ethics committee waived the requirement for informed consent. If patients provided informed written consent to have data from their medical records used in research, please include this information.

3. Please note that PLOS One has specific guidelines on code sharing for submissions in which author-generated code underpins the findings in the manuscript. In these cases, we expect all author-generated code to be made available without restrictions upon publication of the work. Please review our guidelines at https://journals.plos.org/plosone/s/materials-and-software-sharing#loc-sharing-code and ensure that your code is shared in a way that follows best practice and facilitates reproducibility and reuse.

4. Please update your submission to use the PLOS LaTeX template. The template and more information on our requirements for LaTeX submissions can be found at http://journals.plos.org/plosone/s/latex.

5. Thank you for stating the following in the Acknowledgments Section of your manuscript:

“This work was part of the first author's Ph.D. dissertation, completed under the supervision of Effat Alizadeh and Masoud Darabi. This study was supported by the Tabriz University of Medical Sciences, Tabriz, Iran (Grant No. 66664) and the University Medical Center Schleswig-Holstein, Lübeck, Germany.”

We note that you have provided funding information that is not currently declared in your Funding Statement. However, funding information should not appear in the Acknowledgments section or other areas of your manuscript. We will only publish funding information present in the Funding Statement section of the online submission form.

Please remove any funding-related text from the manuscript and let us know how you would like to update your Funding Statement. Currently, your Funding Statement reads as follows:

“This work was part of the first author's Ph.D. dissertation, completed under the supervision of Effat Alizadeh and Masoud Darabi. This study was supported by the Tabriz University of Medical Sciences, Tabriz, Iran (Grant No. 66664) and the University Medical Center Schleswig-Holstein, Lübeck, Germany.”

Please include your amended statements within your cover letter; we will change the online submission form on your behalf.

6. Thank you for stating the following financial disclosure:

“This work was part of the first author's Ph.D. dissertation, completed under the supervision of Effat Alizadeh and Masoud Darabi. This study was supported by the Tabriz University of Medical Sciences, Tabriz, Iran (Grant No. 66664) and the University Medical Center Schleswig-Holstein, Lübeck, Germany.”

Please state what role the funders took in the study.  If the funders had no role, please state: "The funders had no role in study design, data collection and analysis, decision to publish, or preparation of the manuscript."

If this statement is not correct you must amend it as needed.

Please include this amended Role of Funder statement in your cover letter; we will change the online submission form on your behalf.

7. Thank you for stating the following financial disclosure:

“This work was part of the first author's Ph.D. dissertation, completed under the supervision of Effat Alizadeh and Masoud Darabi. This study was supported by the Tabriz University of Medical Sciences, Tabriz, Iran (Grant No. 66664) and the University Medical Center Schleswig-Holstein, Lübeck, Germany.”

We note that one or more of the authors is affiliated with the funding organization, indicating the funder may have had some role in the design, data collection, analysis or preparation of your manuscript for publication; in other words, the funder played an indirect role through the participation of the co-authors. If the funding organization did not play a role in the study design, data collection and analysis, decision to publish, or preparation of the manuscript and only provided financial support in the form of authors' salaries and/or research materials, please do the following:

1. Review your statements relating to the author contributions, and ensure you have specifically and accurately indicated the role(s) that these authors had in your study. These amendments should be made in the online form.

2. Confirm in your cover letter that you agree with the following statement, and we will change the online submission form on your behalf:

“The funder provided support in the form of salaries for authors [insert relevant initials], but did not have any additional role in the study design, data collection and analysis, decision to publish, or preparation of the manuscript. The specific roles of these authors are articulated in the ‘author contributions’ section.

8. PLOS ONE now requires that authors provide the original uncropped and unadjusted images underlying all blot or gel results reported in a submission’s figures or Supporting Information files. This policy and the journal’s other requirements for blot/gel reporting and figure preparation are described in detail at https://journals.plos.org/plosone/s/figures#loc-blot-and-gel-reporting-requirements and https://journals.plos.org/plosone/s/figures#loc-preparing-figures-from-image-files. When you submit your revised manuscript, please ensure that your figures adhere fully to these guidelines and provide the original underlying images for all blot or gel data reported in your submission. See the following link for instructions on providing the original image data: https://journals.plos.org/plosone/s/figures#loc-original-images-for-blots-and-gels.

In your cover letter, please note whether your blot/gel image data are in Supporting Information or posted at a public data repository, provide the repository URL if relevant, and provide specific details as to which raw blot/gel images, if any, are not available. Email us at plosone@plos.org if you have any questions.

9. Your ethics statement should only appear in the Methods section of your manuscript. If your ethics statement is written in any section besides the Methods, please move it to the Methods section and delete it from any other section. Please ensure that your ethics statement is included in your manuscript, as the ethics statement entered into the online submission form will not be published alongside your manuscript.

10. We notice that your supplementary figures are included in the manuscript file. Please remove them and upload them with the file type 'Supporting Information'. Please ensure that each Supporting Information file has a legend listed in the manuscript after the references list.

11. If the reviewer comments include a recommendation to cite specific previously published works, please review and evaluate these publications to determine whether they are relevant and should be cited. There is no requirement to cite these works unless the editor has indicated otherwise.

[Note: HTML markup is below. Please do not edit.]

Reviewers' comments:

Reviewer's Responses to Questions

**Comments to the Author**

1. Is the manuscript technically sound, and do the data support the conclusions?

Reviewer #1: Yes

Reviewer #2: Yes

**********

2. Has the statistical analysis been performed appropriately and rigorously?

Reviewer #1: Yes

Reviewer #2: Yes

**********

3. Have the authors made all data underlying the findings in their manuscript fully available?

The PLOS Data policy

Reviewer #1: Yes

Reviewer #2: Yes

**********

4. Is the manuscript presented in an intelligible fashion and written in standard English?

Reviewer #1: No

Reviewer #2: No

**********

Reviewer #1: The study explores a highly relevant topic in metabolic and regenerative medicine: the paracrine signaling of brown and white adipocytes via exosomes. The use of four different human cell models (OGCs, MCF-7, hADSCs, hUCMSCs) provides a comprehensive overview of how these secretomes influence diverse biological processes, from cancer apoptosis to fertility and stemness. The integration of lipidomic profiling adds significant depth to the phenotypic observations.

The characterization of isolated hADSCs and their subsequent differentiation into BALCs and WALCs is well-documented using flow cytometry, qPCR, and Western Blotting.

The study doesn't limit itself to phenotypic changes but seeks a metabolic explanation through mass spectrometry-based lipidomics. The differential effects (e.g., BALCs-Exo increasing estradiol in OGCs while CM reduces it) are striking and suggest complex regulatory mechanisms.

While the paper establishes what happens, the how remains largely correlative. Identifying specific exosomal cargo (miRNAs or proteins) responsible for the observed effects would significantly strengthen the impact. Moreover, for vesicle characterization, endoplasmic markers such as calreticulin and/or calnexin should be added to exclude contamination of vesicles derived from the endoplasmic reticulum. In figure 7A, change “Annexin 3” with “Annexin V”. Then, the discussion should be more concise, prioritizing the interpretation of key findings and expanding the speculation regarding the potential molecular/proteic content of the vesicles.

Reviewer #2: This manuscript investigates the effects of conditioned media (CM) and exosomes derived from brown adipocyte-like cells (BALCs) and white adipocyte-like cells (WALCs) on different human cell models, including ovarian granulosa cells (OGCs), MCF-7 breast cancer cells, hADSCs, and hUCMSCs. The topic is relevant and potentially interesting because adipocyte-derived extracellular vesicles are increasingly recognized as mediators of intercellular communication. The inclusion of lipidomics also increases the translational value of the study.

However, although the manuscript contains potentially valuable observations, the current version remains descriptive and preliminary.

In particular, the exosome characterization, mechanistic validation, and biological rigor require substantial improvement before the manuscript can be considered suitable for publication.

The authors identify the isolated particles as “exosomes”, but the characterization is incomplete according to current extracellular vesicle (EV) guidelines (MISEV recommendations). Only morphology (TEM/SEM), size, zeta potential, and positive markers (CD9, CD63, TSG101) are shown.The evidence is not sufficient to exclude contamination from proteins, apoptotic bodies, lipoproteins, or other EV populations, especially because a precipitation kit was used for isolation.The authors should add:

- Nanoparticle tracking analysis (NTA) to determine particle concentration and size distribution.

- Negative EV markers (e.g., calnexin, GM130, or albumin) to demonstrate purity.

- Quantification of exosome yield and normalization strategy (particle number or protein amount).

The manuscript assumes that target cells respond directly to adipocyte-derived exosomes, but there is no evidence that exosomes are internalized by recipient cells. The authors must perform exosome uptake assay using fluorescent labeling (e.g., PKH67, DiI, or membrane dye) and microscopy or flow cytometry.

The lipidomics section is interesting but highly speculative. Many conclusions regarding mitochondrial remodeling, oxidative phosphorylation, membrane specialization, or metabolic programming are not experimentally validated. To support these claims, authors should include functional metabolic assays, such as:

- Mitochondrial respiration analysis (Seahorse assay),

- ATP production assay,

- ROS measurements,

- Mitochondrial membrane potential assay.

The osteogenic differentiation conclusion relies mainly on ALP activity. However, ALP is an early marker and does not fully demonstrate osteogenic commitment. The authors should add Alizarin Red quantification and evaluation of osteogenic genes (RUNX2, Osteocalcin, Osteopontin).

English should be revised by a native speaker.

**********

what does this mean?). If published, this will include your full peer review and any attached files.

If you choose “no”, your identity will remain anonymous but your review may still be made public.

**Do you want your identity to be public for this peer review?**  For information about this choice, including consent withdrawal, please see our Privacy Policy

Reviewer #1: No

Reviewer #2: No

**********

[NOTE: If reviewer comments were submitted as an attachment file, they will be attached to this email and accessible via the submission site. Please log into your account, locate the manuscript record, and check for the action link "View Attachments". If this link does not appear, there are no attachment files.]

To ensure your figures meet our technical requirements, please review our figure guidelines: https://journals.plos.org/plosone/s/figures

You may also use PLOS’s free figure tool, NAAS, to help you prepare publication quality figures: https://journals.plos.org/plosone/s/figures#loc-tools-for-figure-preparation.

NAAS will assess whether your figures meet our technical requirements by comparing each figure against our figure specifications.

# Author comment: Author response to Decision Letter 1

**DOI:** 10.1371/journal.pone.0355955.r003

11 Jul 2026

07/07/2026

Manuscript Number: PONE-D-26-16792

Submission of revise,

Dear Professor Editor/s,

Thank you very much for giving us the opportunity to submit a revised version of our manuscript entitled “Brown and white adipocyte like cells derived exosomes differentially regulate stemness, hormone secretion, apoptosis, and lipid metabolism in model cells” for publication in the esteemed Journal PLOS One. We appreciate the time and efforts that editor and the reviewers dedicated to provide feedback on our manuscript and are grateful for the insightful comment on and valuable improvements to our paper. We incorporated the suggestions made by the reviewer. Please see below, for a point-by-point response to the editor/s and reviewer/s comment. Also, we uploaded a revised version changes highlighted/track changed as well as a clean revised manuscript. Thank you in advance. We look forward to hearing from you.

Sincerely,

Dr. Effat Alizadeh,

Associate Professor, Department of Medical Biotechnology,

Faculty of Advanced Medical Sciences, Tabriz University of Medical Sciences, Tabriz, Iran

Phone: +984133341933

Mobile: +989144059856

Alizadehe@tbzmed.ac.ir or e.alizadeh.2010@gmail.com

ORCID codes of Authors:

Zeinab Ghesmati (ORCiD: 0000-0002-8416-2298)

Mohsen Rashid (ORCiD: 0000-0003-2042-1083)

Shabnam Fayezi (ORCiD: 0000-0001-6954-5138)

Effat Alizadeh (ORCiD: 0000-0002-7481-0896)

Masoud Darabi (ORCiD: 0000-0001-6380-272X)

Response to comments:

1.The manuscript is of interest pending some points to be revised, including a more insight on the specific exosomal components driving these changes and vesicle characterization by assessing an endoplasmic reticulum marker (such as Calnexin or Calreticulin) to rule out cellular contamination. This will make more robust the findings

Response: We thank the Academic Editor for this valuable suggestion. The present study was designed as an in vitro functional and lipidomic analysis of adipocyte-derived exosome-enriched preparations from brown and white adipocyte-like cells, rather than a comprehensive molecular cargo discovery study of exosomes. We have now acknowledged this limitation in the Discussion (Page number: 39) and added that future proteomic and transcriptomic analyses, including miRNA profiling, will be required to identify candidate molecular mediators and validate their functional contribution.

Calnexin, as a negative marker, has now been included in the additional characterization experiments for both groups (BALC Exo and WALC Exo) by Western blotting. The revised Methods (Page number: 12), Results, and Figure 5G (Page numbers: 21-22) now indicate that the isolated exosome preparations were positive for CD9, CD63, and TSG101 and negative for calnexin.

Journal Requirements:

1. Please ensure that your manuscript meets PLOS ONE's style requirements, including those for file naming. The PLOS ONE style templates can be found at

https://journals.plos.org/plosone/s/file?id=wjVg/PLOSOne_formatting_sample_main_body.pdf and https://journals.plos.org/plosone/s/file?id=ba62/PLOSOne_formatting_sample_title_authors_affiliations.pdf

Response: Thank you for this comment. We have carefully reviewed the manuscript and supporting files to ensure compliance with PLOS ONE style requirements, including file naming conventions and formatting guidelines. The manuscript and associated files have been revised accordingly and prepared in accordance with the PLOS ONE style templates.

2. Please provide additional details regarding participant consent. In the ethics statement in the Methods and online submission information, please ensure that you have specified (1) whether consent was informed and (2) what type you obtained (for instance, written or verbal, and if verbal, how it was documented and witnessed). If your study included minors, state whether you obtained consent from parents or guardians. If the need for consent was waived by the ethics committee, please include this information. If you are reporting a retrospective study of medical records or archived samples, please ensure that you have discussed whether all data were fully anonymized before you accessed them and/or whether the IRB or ethics committee waived the requirement for informed consent. If patients provided informed written consent to have data from their medical records used in research, please include this information.

Response:

Thank you very much for this important comment. We have revised the Ethics Statement in the Methods section to provide additional details regarding participant consent. Specifically, we clarified that informed consent was obtained from all participants prior to sample collection and study participation. We also specified the type of consent obtained, indicating that written informed consent was collected in accordance with the requirements of the approving ethics committee. Participation was voluntary, and all procedures complied with institutional guidelines and the principles of the Declaration of Helsinki. Where applicable, we additionally clarified that no minors were included in this study; therefore, parental or guardian consent was not required. No waiver of informed consent was requested or granted by the ethics committee. The revised ethics statement has been updated accordingly in the manuscript.

3. Please note that PLOS One has specific guidelines on code sharing for submissions in which author-generated code underpins the findings in the manuscript. In these cases, we expect all author-generated code to be made available without restrictions upon publication of the work. Please review our guidelines at https://journals.plos.org/plosone/s/materials-and-software-sharing#loc-sharing-code and ensure that your code is shared in a way that follows best practice and facilitates reproducibility and reuse.

Response: Thank you for bringing this to our attention. We reviewed the PLOS ONE guidelines regarding code sharing and confirm that no author-generated code was developed or used to generate, process, analyze, or interpret the findings presented in this manuscript. All analyses were performed using standard commercially available and/or publicly accessible software with their built-in functions and settings. Accordingly, there is no custom code associated with this study that requires public deposition.

4. Please update your submission to use the PLOS LaTeX template. The template and more information on our requirements for LaTeX submissions can be found at http://journals.plos.org/plosone/s/latex.

Response: Thank you for your comment and for providing the PLOS formatting guidance. We understand the recommendation regarding use of the PLOS LaTeX template. However, we do not have an existing LaTeX version of the manuscript available. Therefore, we have retained the manuscript in its current format and ensured that it complies with all applicable journal formatting and submission requirements.

5. Thank you for stating the following in the Acknowledgments Section of your manuscript:

“This work was part of the first author's Ph.D. dissertation, completed under the supervision of Effat Alizadeh and Masoud Darabi. This study was supported by the Tabriz University of Medical Sciences, Tabriz, Iran (Grant No. 66664) and the University Medical Center Schleswig-Holstein, Lübeck, Germany.” We note that you have provided funding information that is not currently declared in your Funding Statement. However, funding information should not appear in the Acknowledgments section or other areas of your manuscript. We will only publish funding information present in the Funding Statement section of the online submission form. Please remove any funding-related text from the manuscript and let us know how you would like to update your Funding Statement. Currently, your Funding Statement reads as follows: “This work was part of the first author's Ph.D. dissertation, completed under the supervision of Effat Alizadeh and Masoud Darabi. This study was supported by the Tabriz University of Medical Sciences, Tabriz, Iran (Grant No. 66664) and the University Medical Center Schleswig-Holstein, Lübeck, Germany.”Please include your amended statements within your cover letter; we will change the online submission form on your behalf.

Response:

We greatly appreciate your clarification regarding the presentation of funding information. We acknowledge that funding-related information was inadvertently included in the manuscript text. In accordance with PLOS ONE requirements, we have removed all funding-related statements from the manuscript and retained only the non-funding acknowledgment text.We request that the Funding Statement in the online submission system be amended to include only the funding information as follows:

“This study was supported by the Tabriz University of Medical Sciences, Tabriz, Iran (Grant No. 66664) and the University Medical Center Schleswig-Holstein, Lübeck, Germany.”

The following statement has been retained in the manuscript Acknowledgments section without amendment, as it is not a funding declaration:

“This work was part of the first author's Ph.D. dissertation, completed under the supervision of Effat Alizadeh and Masoud Darabi.”

Please update the online submission form accordingly.

6. Thank you for stating the following financial disclosure:

“This work was part of the first author's Ph.D. dissertation, completed under the supervision of Effat Alizadeh and Masoud Darabi. This study was supported by the Tabriz University of Medical Sciences, Tabriz, Iran (Grant No. 66664) and the University Medical Center Schleswig-Holstein, Lübeck, Germany.” Please state what role the funders took in the study. If the funders had no role, please state: "The funders had no role in study design, data collection and analysis, decision to publish, or preparation of the manuscript." If this statement is not correct you must amend it as needed. Please include this amended Role of Funder statement in your cover letter; we will change the online submission form on your behalf.

Response: Many thanks to request for clarification regarding the role of the funders.

We confirm that the funding organizations provided financial support for conducting the study but had no involvement in the scientific or publication-related aspects of the work. Accordingly, we request that the Role of Funder statement in the online submission system be updated as follows: “The funders had no role in study design, data collection and analysis, decision to publish, or preparation of the manuscript.” Please update the online submission form accordingly.

7. Thank you for stating the following financial disclosure:

“This work was part of the first author's Ph.D. dissertation, completed under the supervision of Effat Alizadeh and Masoud Darabi. This study was supported by the Tabriz University of Medical Sciences, Tabriz, Iran (Grant No. 66664) and the University Medical Center Schleswig-Holstein, Lübeck, Germany.” We note that one or more of the authors is affiliated with the funding organization, indicating the funder may have had some role in the design, data collection, analysis or preparation of your manuscript for publication; in other words, the funder played an indirect role through the participation of the co-authors. If the funding organization did not play a role in the study design, data collection and analysis, decision to publish, or preparation of the manuscript and only provided financial support in the form of authors' salaries and/or research materials, please do the following:

1. Review your statements relating to the author contributions, and ensure you have specifically and accurately indicated the role(s) that these authors had in your study. These amendments should be made in the online form.

2. Confirm in your cover letter that you agree with the following statement, and we will change the online submission form on your behalf:

“The funder provided support in the form of salaries for authors [insert relevant initials], but did not have any additional role in the study design, data collection and analysis, decision to publish, or preparation of the manuscript. The specific roles of these authors are articulated in the ‘author contributions’ section.

Response: Thank you for your clarification regarding disclosure of the funder’s role and author affiliations. We have reviewed the Author Contributions statements and ensured that the contributions of all authors are specifically and accurately reflected in the online submission information. We confirm that the funding organizations provided support solely in the form of research funding. Furthermore, the funding organizations had no additional role in the study design, data collection and analysis, decision to publish, or preparation of the manuscript. Any contributions made by authors affiliated with the funding organizations were performed independently in their capacity as researchers and are explicitly described in the Author Contributions section. Accordingly, we request that the online submission form be updated to reflect this clarification.

8. PLOS ONE now requires that authors provide the original uncropped and unadjusted images underlying all blot or gel results reported in a submission’s figures or Supporting Information files. This policy and the journal’s other requirements for blot/gel reporting and figure preparation are described in detail at https://journals.plos.org/plosone/s/figures#loc-blot-and-gel-reporting-requirements and https://journals.plos.org/plosone/s/figures#loc-preparing-figures-from-image-files. When you submit your revised manuscript, please ensure that your figures adhere fully to these guidelines and provide the original underlying images for all blot or gel data reported in your submission. See the following link for instructions on providing the original image data: https://journals.plos.org/plosone/s/figures#loc-original-images-for-blots-and-gels. In your cover letter, please note whether your blot/gel image data are in Supporting Information or posted at a public data repository, provide the repository URL if relevant, and provide specific details as to which

Response:

Many thanks, we have reviewed the journal guidelines and ensured that all figures included in the revised submission comply with the requirements for blot/gel reporting and figure preparation. The original uncropped and unadjusted images underlying all blot and gel results reported in the manuscript have been provided as Supporting Information files to facilitate transparency, reproducibility, and assessment of image integrity. No modifications beyond those described in the manuscript were applied to the original source images.

9. Your ethics statement should only appear in the Methods section of your manuscript. If your ethics statement is written in any section besides the Methods, please move it to the Methods section and delete it from any other section. Please ensure that your ethics statement is included in your manuscript, as the ethics statement entered into the online submission form will not be published alongside your manuscript.

Response: We have reviewed the manuscript and ensured that the ethics statement appears only within the Methods section in accordance with PLOS ONE requirements. Any duplicate ethics-related text appearing elsewhere in the manuscript has been removed.

10. We notice that your supplementary figures are included in the manuscript file. Please remove them and upload them with the file type 'Supporting Information'. Please ensure that each Supporting Information file has a legend listed in the manuscript after the references list.

Response: Thank you for bringing this to our attention. We have removed the supplementary figures from the main manuscript file and uploaded them separately under the appropriate file type, “Supporting Information,” in accordance with the journal requirements. Additionally, legends for each Supporting Information file have been added to the manuscript and placed after the references section as requested.

11. If the reviewer comments include a recommendation to cite specific previously published works, please review and evaluate these publications t

Attachment Submitted filename: Response to Reviewer-Editor comments.docx

# Peer review: Decision Letter 1

**DOI:** 10.1371/journal.pone.0355955.r004

**Authors:** Mohammad H. Ghazimoradi

28 Jul 2026

Brown and white adipocyte like cells derived exosomes differentially regulate stemness, hormone secretion, apoptosis, and lipid metabolism in model cells

PONE-D-26-16792R1

Dear Dr. Alizadeh,

We’re pleased to inform you that your manuscript has been judged scientifically suitable for publication and will be formally accepted for publication once it meets all outstanding technical requirements.

Within one week, you’ll receive an e-mail detailing the required amendments. When these have been addressed, you’ll receive a formal acceptance letter and your manuscript will be scheduled for publication.

An invoice will be generated when your article is formally accepted. Please note, if your institution has a publishing partnership with PLOS and your article meets the relevant criteria, all or part of your publication costs will be covered. Please make sure your user information is up-to-date by logging into Editorial Manager at Editorial Manager® and clicking the ‘Update My Information' link at the top of the page. For questions related to billing, please contact billing support.

If your institution or institutions have a press office, please notify them about your upcoming paper to help maximize its impact. If they’ll be preparing press materials, please inform our press team as soon as possible -- no later than 48 hours after receiving the formal acceptance. Your manuscript will remain under strict press embargo until 2 pm Eastern Time on the date of publication. For more information, please contact onepress@plos.org.

Kind regards,

Mohammad H. Ghazimoradi

Academic Editor

PLOS One

**Comments to the Author**

Reviewer #1: All comments have been addressed

Reviewer #2: All comments have been addressed

**********

2. Is the manuscript technically sound, and do the data support the conclusions?

Reviewer #1: Yes

Reviewer #2: (No Response)

**********

3. Has the statistical analysis been performed appropriately and rigorously?

Reviewer #1: Yes

Reviewer #2: (No Response)

**********

4. Have the authors made all data underlying the findings in their manuscript fully available?

The PLOS Data policy

Reviewer #1: Yes

Reviewer #2: (No Response)

**********

5. Is the manuscript presented in an intelligible fashion and written in standard English?

Reviewer #1: Yes

Reviewer #2: (No Response)

**********

Reviewer #1: (No Response)

Reviewer #2: (No Response)

**********

what does this mean?). If published, this will include your full peer review and any attached files.

If you choose “no”, your identity will remain anonymous but your review may still be made public.

**Do you want your identity to be public for this peer review?**  For information about this choice, including consent withdrawal, please see our Privacy Policy

Reviewer #1: No

Reviewer #2: No

**********

# Editor report: Acceptance letter

**DOI:** 10.1371/journal.pone.0355955.r005

**Authors:** Mohammad H. Ghazimoradi

PONE-D-26-16792R1

PLOS One

Dear Dr. Alizadeh,

I'm pleased to inform you that your manuscript has been deemed suitable for publication in PLOS One. Congratulations! Your manuscript is now being handed over to our production team.

At this stage, our production department will prepare your paper for publication. This includes ensuring the following:

* All references, tables, and figures are properly cited

* All relevant supporting information is included in the manuscript submission,

* There are no issues that prevent the paper from being properly typeset

You will receive further instructions from the production team, including instructions on how to review your proof when it is ready. Please keep in mind that we are working through a large volume of accepted articles, so please give us a few days to review your paper and let you know the next and final steps.

Lastly, if your institution or institutions have a press office, please let them know about your upcoming paper now to help maximize its impact. If they'll be preparing press materials, please inform our press team within the next 48 hours. Your manuscript will remain under strict press embargo until 2 pm Eastern Time on the date of publication. For more information, please contact onepress@plos.org.

You will receive an invoice from PLOS for your publication fee after your manuscript has reached the completed accept phase. If you receive an email requesting payment before acceptance or for any other service, this may be a phishing scheme. Learn how to identify phishing emails and protect your accounts at https://explore.plos.org/phishing.

If we can help with anything else, please email us at customercare@plos.org.

Thank you for submitting your work to PLOS ONE and supporting open access.

Kind regards,

PLOS ONE Editorial Office Staff

on behalf of

Dr. Mohammad H. Ghazimoradi

Academic Editor

PLOS One
